# Integrated cross-sectoral surveillance of antimicrobial resistance genotypes and phenotypes across disparate reservoirs

**DOI:** 10.1128/msystems.00741-26

**Published:** 2026-09-28

**Authors:** Thomas D. Watts, Thanavit Jirapanjawat, Laura Perlaza-Jiménez, Francesco Ricci, Eve Tudor-Matthew, Eleonora Chiri, Sean K. Bay, Rhys Grinter, Rachael Lappan, Trevor Lithgow, Laura C. Woods, Chris Greening

**Affiliations:** 1 Infection Program, Biomedicine Discovery Institute and Department of Microbiology, Monash University 2541, Clayton, Victoria, Australia; 2 Centre to Impact AMR, Monash University 2541, Clayton, Victoria, Australia; Northwestern University, Evanston, Illinois, USA; Michigan Technological University, Houghton, Michigan, USA; Agriculture and Agri-Food Canada Lethbridge Research and Development Centre, Lethbridge, Alberta, Canada

**Keywords:** antibiotic resistance, surveillance, environmental microbiology, metagenomics, qPCR, bacterial isolation

## Abstract

Antimicrobial-resistant (AMR) bacteria and genes are continually exchanged among humans, animals, and environmental reservoirs. Disparate and siloed surveillance methods present a major challenge for tracing and disrupting AMR transmission, with clinical monitoring focusing on detecting specific pathogens or specific genes of interest, and environmental surveys often relying on inferences drawn from indicator organisms. Here, we demonstrate that, following sample-specific pre-processing, common surveillance approaches can be applied consistently to profile AMR abundance, distribution, and phenotypes across diverse reservoirs in both urban and agricultural settings. Across all sample types, three core methods provided complementary insights: (i) quantitative PCR (qPCR) arrays to measure multiple AMR genes, (ii) gene- and genome-centric metagenomics for comprehensive resistome profiling, and (iii) culture-based genomics with susceptibility testing to link genotypes to phenotypes. We applied this approach to profile 1,032 metagenome-assembled genomes, 66 bacterial isolate genomes, and 78 and 6,642 AMR genes/reference sequences via qPCR and metagenomics, respectively. This integrated framework revealed the prevalence and diversity of resistance mechanisms in both putative pathogens and non-pathogenic bacteria with potentially transmissible genes, with wastewater especially enriched in AMR genes. We detected mismatches between genotype and phenotype predictions and a prevalence of intermediate resistance phenotypes, highlighting how many mechanisms of environmental resistance remain poorly understood. Overall, this study demonstrates that unified, field-leading surveillance methods can be used in diverse environmental and animal samples while highlighting that multiple methods are needed to capture the diverse AMR genotypes and phenotypes in these settings to enable comprehensive monitoring and adaptive solutions to restrict transmission.

**IMPORTANCE:**

Antimicrobial resistance (AMR) is driven by the exchange of resistant bacteria and genes across interconnected human, animal, and environmental reservoirs, yet fragmented surveillance limits our ability to track transmission and intervene effectively. We demonstrate that widely used surveillance methods can be applied cohesively across diverse sample types, including wastewater, soil, water, agricultural environments, and fecal samples, to generate comparable insights into AMR abundance, diversity, and phenotype. By integrating qPCR, metagenomics, and culture-based genomics, we reveal resistance in both pathogenic and non-pathogenic bacteria, reaffirm wastewater as a major AMR reservoir, and uncover frequent mismatches between genetic predictions and observed susceptibility. The prevalence of intermediate resistance further suggests that many environmental resistance mechanisms remain poorly understood. These findings show that no single method captures AMR complexity and highlight the need for unified, multi-method surveillance to support comprehensive monitoring and strategies to control the spread of resistance.

## INTRODUCTION

Without intervention, antimicrobial resistance (AMR)-associated infections are predicted to cause up to 10 million deaths annually by 2050, alongside a trillion-dollar economic burden on global healthcare systems (1, 2). AMR is quintessentially a One Health issue, with resistance determinants and resistant bacteria consistently exchanged between humans, animals, and the environment (3, 4). The concept of resistome describes all the resistance genes present in a given microbial community (5). Assessing the sources and impacts of environmental resistomes is difficult, given the natural diversity of antimicrobial resistance genes (ARGs) in the environment, making it hard to interpret what is “normal” and what is indicative of a problem such as imminent spillover to another reservoir. Additionally, resistance may emerge due to selective pressures enriching ARGs in resident microbiota, contamination with bacteria from human or animal sources that harbor existing resistance genes, or a combination of both (3, 6). For example, in many countries, antibiotics used in human medicine are also extensively applied in livestock production (7–9), promoting AMR in animals with the potential for cross-reservoir transmission. Resistant bacteria are also released into the wider environment via wastewater discharge, septic leakage, stormwater runoff, animal feces, and agricultural runoff (10, 11). Urban-adjacent water systems can act as important reservoirs and transmission routes, particularly in low- to middle-income settings (12). Sub-lethal concentrations of antibiotics from agricultural, hospital, or wastewater sources also act as selective pressures in these systems, fostering the persistence and diversification of resistant populations (13).

In these environments, AMR can occur through acquisition of mutations or the acquisition of ARGs via horizontal gene transfer mediated by mobile genetic elements or transformation. Horizontal gene transfer has the potential to embed resistance traits in resident microbial communities, or spread ARGs from environmental bacteria to clinically relevant human pathogens (14–16). Bacteria with broad niches, such as drug-resistant *Klebsiella pneumoniae,* have been highlighted as conduits allowing environmental ARGs to enter clinical settings (14). Importantly, environmental and agricultural settings are not only passive sinks but active incubators of resistance, where several clinically important ARGs, including plasmid-derived *mcr-1* (colistin resistance), were first identified in animals before becoming widespread in the clinic (17). Identifying hotspots of ARG diversity and abundance is also critical for understanding the emergence of novel resistance genes (6, 18).

Despite the importance of environmental reservoirs, AMR surveillance remains disproportionately pathogen-centric and biased toward clinical settings. The most recent Antimicrobial Usage and Resistance in Australia report highlights that surveillance still focuses on clinical and aged-care facilities, focusing only on AMR pathogen detection (19). In addition, environmental surveillance often relies on indicator organisms such as *Escherichia coli* or *Enterococci* to signal fecal contamination, representing a narrow approach that does not adequately reflect the diversity and abundance of pathogens or AMR in environmental niches (20–22). Clinical AMR monitoring is tightly regulated and globally coordinated; by contrast, approaches for environmental surveillance vary, with a range of sample types necessitating tailored approaches. Detection methods are diverse; there is limited agreement on which organisms and ARGs should be prioritized, and no global standardized frameworks exist (23). Furthermore, many surveillance efforts use either genotypic or phenotypic methods in isolation. However, as we recently reviewed (4), different techniques each have complementary strengths and limitations: targeted qPCR enables sensitive detection of known ARGs, but cannot identify novel variants; metagenomics provides broad gene discovery, but limited resolution of host context; and culture-based isolation and genomics link ARGs to phenotypic resistance, but capture only a fraction of microbial diversity in an environment. Additionally, linking ARG detection to clinically relevant phenotypes is also important. Gene carriage does not always translate to resistance, and without confirming phenotype, the clinical significance of environmental AMR can be uncertain. A unified, cross-sectoral surveillance framework that integrates complementary methods is therefore essential to move beyond documenting AMR and toward building actionable systems to inform mitigation and intervention strategies across the One Health spectrum (4, 23–25).

Here, we demonstrate the feasibility and utility of an integrated approach to investigate the distribution of AMR across diverse environmental reservoirs within the greater Melbourne metropolitan area. Using coordinated methodologies including qPCR, metagenomics, bacterial isolation, antibiotic susceptibility testing, and whole-genome sequencing (WGS), we generate a comprehensive view of ARG carriage and resistance phenotypes across soil, water, and fecal samples. This unified framework enables high-resolution identification of environmental AMR and helps inform future surveillance strategies that can be applied in a range of contexts.

## RESULTS

### Antibiotic resistance genes and pathogenic genera are present within disparate environmental sources across Melbourne, Australia

**Fig 1:**
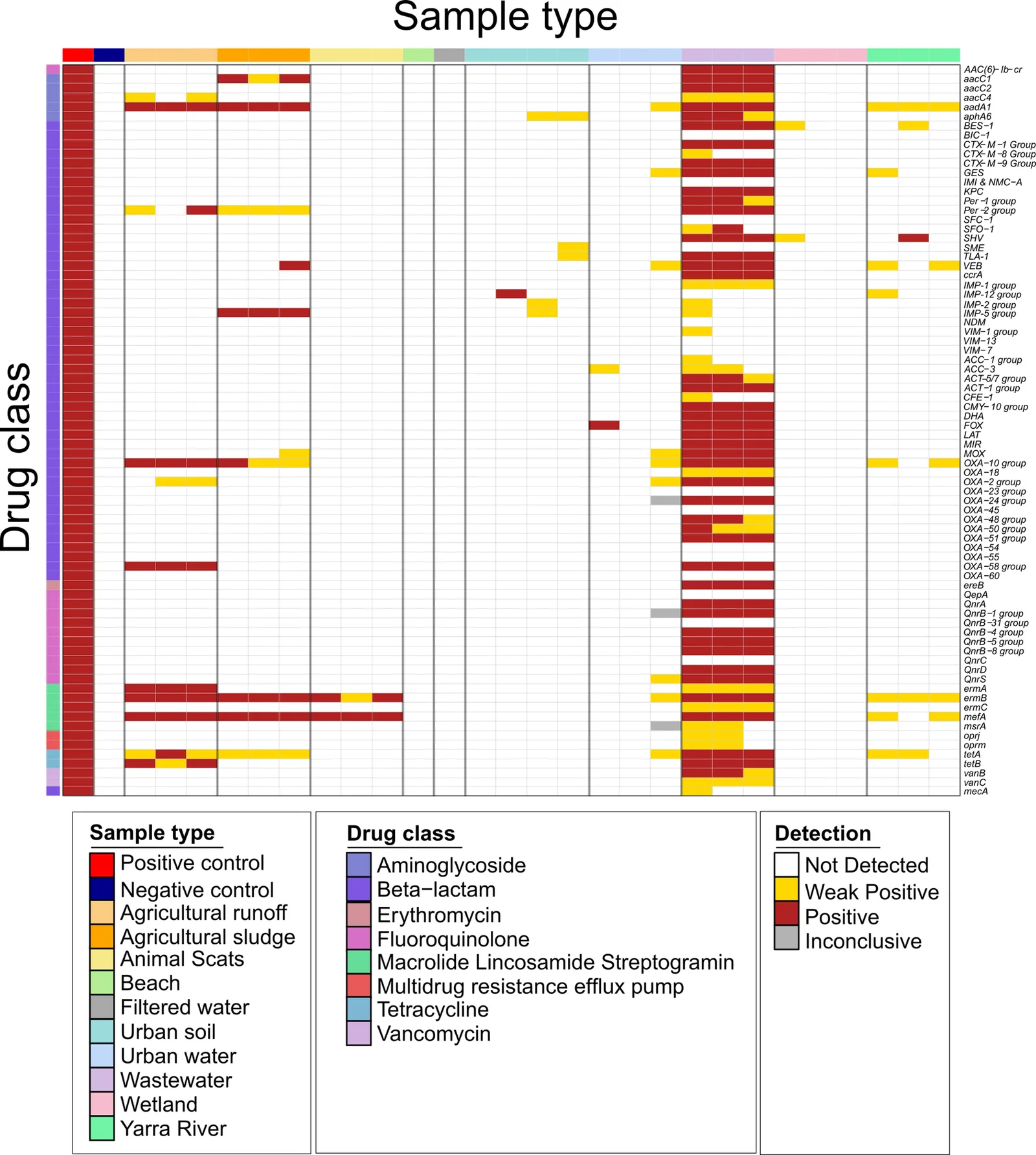
ARG detection by qPCR array. DNA extracts were applied to the QIAGEN Microbial DNA qPCR array for antibiotic resistance genes, which encompasses a range of ARGs. A “±” result indicates weak detection (qPCR threshold value within 3–6 cycles of the no-template control).

We sought to understand the distribution of AMR pathogens in the environment surrounding Melbourne and to compare different surveillance approaches. Nine diverse sample types were selected, including agricultural runoff and sludge collected from a dairy farm, wetland water, urban park soil and water, animal scats, wastewater, urban beach sediment, and river water (Table S1 and Fig. S1). Samples were collected and pre-processed differently depending on sample type, but all subsequent procedures, including DNA extraction and downstream analysis, were conducted using the same pipeline. To assess the distribution of ARGs within the different environments, we performed qPCR arrays to detect 78 genes associated with AMR or virulence (Fig. 1). Wastewater is a rich environmental sink where microbes from diverse sources converge, and the highest diversity of ARGs was associated with this sample type (average 55.6 ARGs). Genes from almost all antimicrobial classes tested by the qPCR were represented in wastewater samples, including determinants of beta-lactam resistance (*bla_OXA_*, *bla_SHV_*, *bla_KPC_*, *bla_IMP_*, *bla_VIM_*, *bla_NDM_*, and *bla_CTX-M_*), vancomycin resistance (*vanA* and *vanB*), erythromycin resistance (*ermA*, *ermB*, and *ermC*), tetracycline resistance (*tetA* and *tetB*), and fluoroquinolone resistance (*qnrA*, *qnrB*, *qnrD*, and *qnrS*) (Fig. 1).

ARGs were broadly distributed in other samples, although there were far fewer ARGs detected per sample (average 3.6) compared to wastewater (average 55.6). Agricultural samples showed ARGs associated with tetracycline resistance, macrolide resistance, beta-lactam resistance, and aminoglycoside resistance. Animal scats collected from an urban park were also positive for the determinants of macrolide resistance. The single beach sample tested was negative for all ARGs tested with qPCR, whereas urban park soil and water contained determinants of aminoglycoside resistance and beta-lactam resistance. Wetland and river samples were positive for aminoglycoside, macrolide, beta-lactam, and tetracycline resistance determinants.

### Metagenomic data sets mirror AMR distribution highlighted by qPCR arrays and link ARGs to specific metagenome-assembled genomes

**Fig 2:**
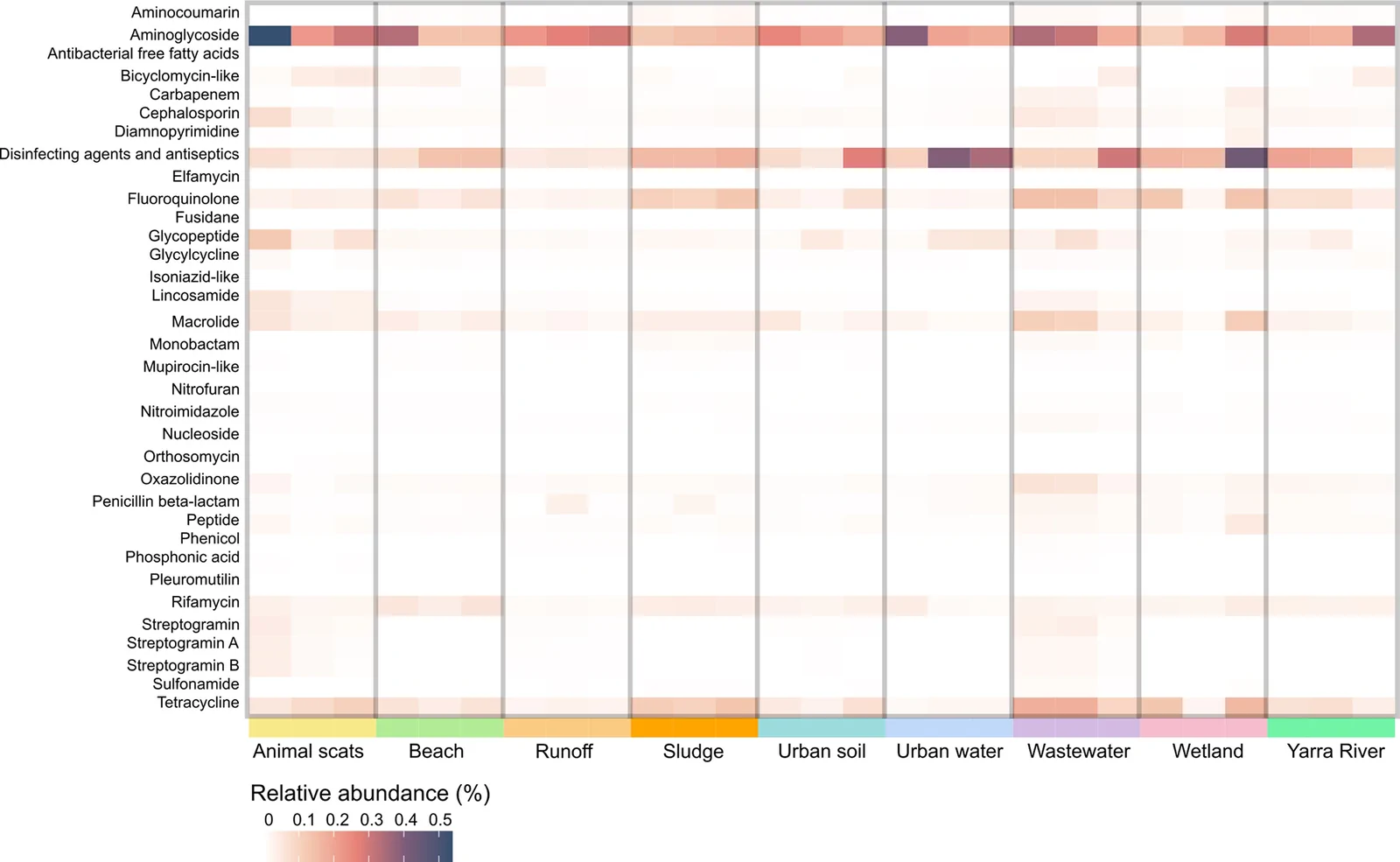
Heatmap depicting ARG abundance in metagenome reads with the CARD RGI. Metagenome short reads for each sample type (*n* = 3) were screened with RGI to determine ARG carriage. Detected ARGs were collapsed to drug class, and relative abundance (%) was calculated for each sample.

Although qPCR rapidly reports the diversity of known ARGs that are present in the environment, it cannot identify ARGs other than those included in the array. qPCR also has limited capacity to link ARGs to specific hosts, which is important for understanding emerging resistance and reservoirs. We therefore conducted metagenomic sequencing (NextSeq2000) on each sample to complement qPCR surveillance, generating an average of ~120 million paired reads per sample. Identical methods for library preparation, sequencing, and downstream analysis were used for all samples. Quality-controlled reads from each metagenome data set were used to query the Comprehensive Antibiotic Resistance Database (CARD) (implemented via the resistance gene identifier (RGI) tool) to determine the breadth of ARGs present (Fig. 2). This catch-all approach yielded a more comprehensive picture of the total resistome of these samples, including both chromosomally encoded and plasmid-encoded resistance genes. All samples had a rich diversity of ARG classes, with wastewater samples displaying the highest number of drug classes and the highest average abundance of ARGs (~32–33 classes, average abundance of ARGs in the community 1.2% ± 0.2% SD) (Tables S2 and S3). The increase in the number of ARG classes identified for each sample likely reflects the enhanced breadth of detection afforded by using a more comprehensive database (6,642 genes). This is especially clear when compared with the targeted approach of qPCR (78 genes), where the specific primers used in the qPCR array preclude the capture of the full diversity of sequences within each ARG group. Putative resistance to aminoglycosides, disinfectants and antiseptics, tetracycline, fluoroquinolone, and glycopeptide was both widespread and highly abundant in all sample types. However, this finding likely reflects the environmental role of the genes that confer resistance to these antibiotics, rather than an active AMR risk. Additionally, the abundance of fluoroquinolones, aminoglycosides, and disinfectant-associated categories is largely influenced by genes encoding regulators and efflux systems, which likely do not explicitly confer clinical resistance but may contribute to reduced susceptibility. For example, putative aminoglycoside and disinfectant-associated agent resistance appeared to be primarily due to the abundance of osmotic stress-related response regulators (*kdpE*) and efflux pumps (*qacG*) (Table S3). It should be emphasized that caution should be taken when attributing ARG abundance to AMR risk, especially when many of the genes detected by RGI likely reflect intrinsic ecological processes rather than active resistance. A similar distribution of beta-lactamase variants (*bla_OXA_*, *bla_SHV_*, *bla_KPC_*, *bla_IMP_*, *bla_VIM_*, *bla_NDM_*, and *bla_CTX-M_*) was detected in metagenomic data compared to qPCR arrays, where wastewater samples showed higher levels of these genes, likely reflecting the abundance of human fecal bacteria present within these samples (Table S3).

To better link ARGs/AMR to specific community members and identify potential emerging AMR pathogens or reservoirs, metagenomes within each environment were co-assembled and binned to yield 1,032 species level (0.95 average nucleotide identity [ANI]) metagenome-assembled genomes (MAGs). MAG coverage and relative abundance varied depending on the sample, with the number of unmapped reads ranging between 40% and 96% (Tables S4 and S5). Taxonomic classification of MAGs showed that they span 27 bacterial phyla encompassing Pseudomonadota/Proteobacteria (24.7%), Bacteroidota (19.9%), Bacilliota/Firmicutes (19.5%), and Actinomycetota (17.3%) (Table S6). The majority of MAGs appeared to represent autochthonous organisms commonly associated with their environment, although putative opportunistic human pathogens were identified in a subset of samples (Table S6), including *Campylobacter upsaliensis* (animal scats) (26), *Escherichia coli* (beach sand, constructed wetland, river), and *Aeromonas sobria* (27) (urban water). Genomes of several other potential pathogens were also reconstructed from wastewater, namely *Streptococcus suis* (28), *Sutterella wadsworthensis* (29), *Bilophila wadsworthia* (30), *Laribacter hongkongensis* (31, 32)*,* and *Aliarcobacter butzleri* (33). Virulence factor screening and sequence typing showed that the *E. coli* MAGs were from clonal complex ST73 and possessed a variety of virulence factors, including type I fimbriae (Table S7). ST73 complex *E. coli* are associated with urinary tract infection in humans (34, 35). The other organisms reported are rare opportunistic pathogens; therefore, the virulence factors and sequence types associated with pathogenic strains are less well characterized.

**Fig 3:**
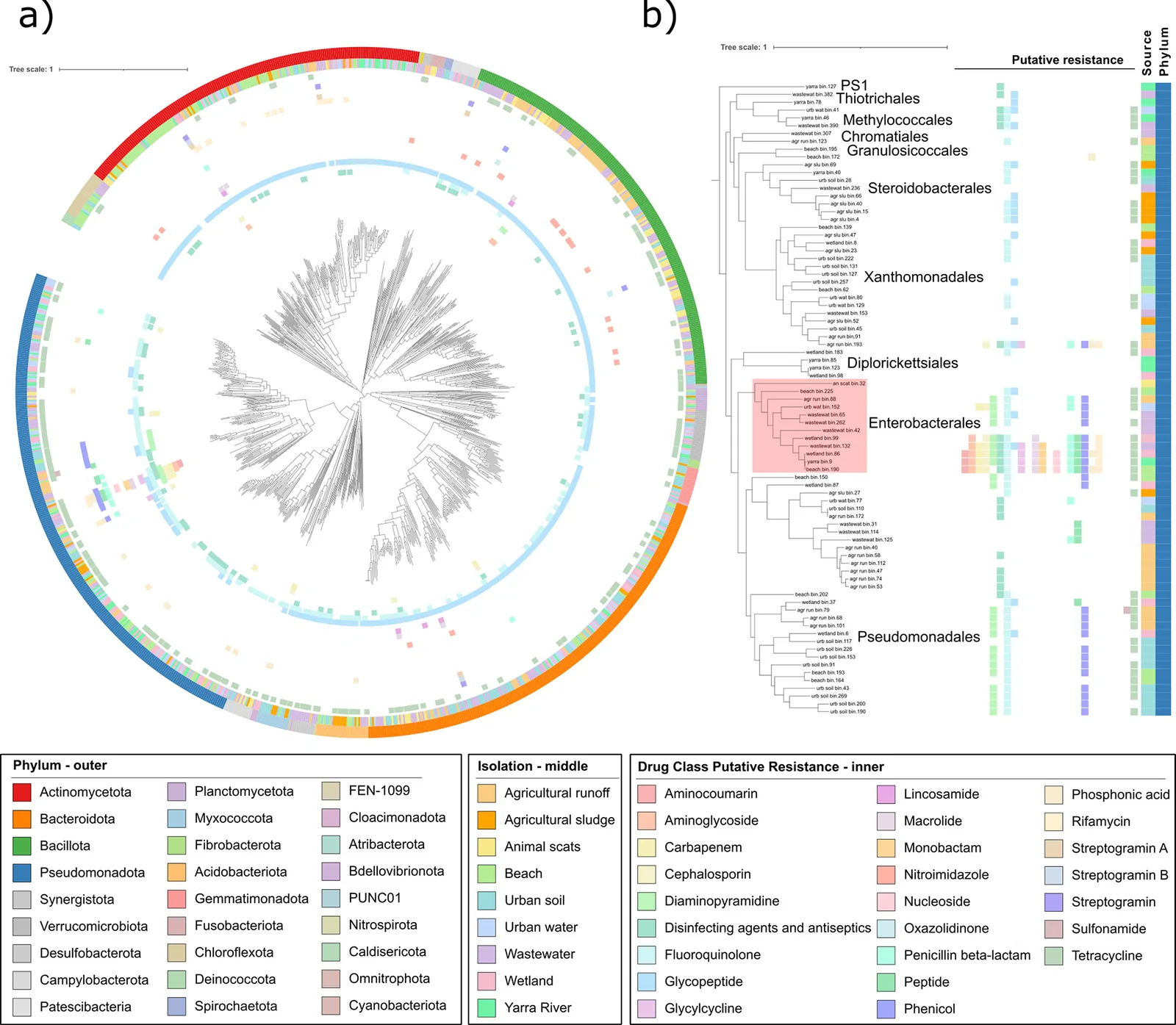
ARGs show a widespread distribution within MAGs but are prevalent within Pseudomonadota. (**a**) Maximum-likelihood phylogenetic tree of 1,032 environmental MAGs reconstructed from samples taken across Melbourne, Australia. The outermost ring indicates phyla, the middle ring indicates isolation source, and the innermost rings indicate drug resistance determined via comparison to the CARD database with RGI. (**b**) A zoomed inset showing Pseudomonadota MAGs with high ARG carriage.

MAGs were screened for chromosomally encoded ARGs using comprehensive databases (CARD), which were subsequently collapsed into AMR classes and used to adorn a maximum-likelihood phylogenetic tree (Fig. 3a). ARGs were present in each environment and were not confined to a single phylum (27 phyla). Genes associated with reduced susceptibility and putative resistance to glycopeptides (*vanG, vanH, vanO, vanT, vanW,* and *vanY*) (*n* = 619, 60%), tetracyclines (*tetA, tetB, adeF, tetQ, tetZ, otrA, poxtA,* and *tet* [36]) (*n* = 231, 22.4%), fluoroquinolones (*adeF, marA, soxS, soxR, sdrM, rsmA, acrA, acrB, acrE, acrF, acrS, emrA, emrB, emrR, mdtH,* and *patB*) (*n* = 201, 19.5%), and disinfecting agents/antiseptics (*qacG*, *qacJ*, and *qacL*) (*n* = 79, 7.7%) were most prevalent within MAGs, including numerous non-pathogenic bacteria (Fig. 3a; Tables S6 and S8). Many of these genes encode transcriptional regulators, two-component systems, or efflux pumps that are associated with reduced susceptibility and clinical AMR only when paired with other resistance mechanisms; therefore, their contribution to clinical resistance should be approached with caution. These genes are also important for stress responses and other ecological processes, and their abundance likely primarily reflects their importance for environmental survival, rather than active AMR risk. This was specifically observed for putative fluoroquinolone resistance where many of the genes identified are resistance associated (*adeF, marA, soxS, soxR, sdrM, rsmA, acrA, acrB, acrE, acrF, acrS, emrA, emrB, emrR, mdtH,* and *patB)* rather than explicitly conferring resistance (*qnr genes, aac(6′)-Ib-cr, qepA, oqxAB,* or mutations in *gyrA, gyrB, parC,* or *parE*). Furthermore, we noted that many glycopeptide-resistance-associated gene hits (*vanG, vanH, vanO, vanT, vanW,* and *vanY*) showed between ~22% and 76% amino acid identity (Table S8) and were not always encoded as part of a *van* resistance cluster, suggesting that these ARGs likely do not confer resistance. Therefore, both putative glycopeptide and fluoroquinolone resistance should be interpreted cautiously in these environmental resistomes. This reinforces the idea that ARG abundance is not entirely indicative of AMR risk, particularly when AMR-associated determinants likely represent underlying ecological processes.

Prevalence of carbapenem and cephalosporin resistance genes was more limited (*n* = 9, 0.9% and *n* = 12, 1.2%, respectively), but still detected among both autochthonous bacteria (*Mangrovibacter* spp., *Flavobacterium* spp., *Qipengyuania* spp., *Pantoea* spp., *Phocaeicola vulgatus*, *Bacteroides uniformis*, *Luteimonas* spp*.*, and *Rhodoferax* spp.) and putative pathogens (*E. coli*, *L. hongkongensis*, and *A. sobria*). Pseudomonadota MAGs had the most AMR diversity, with some genomes harboring genes putatively conferring resistance to up to 17 different antibiotics (Fig. 3b). Notably, three *E. coli* MAGs from beach, wetland, and river sources possessed putative resistance to 16–17 antibiotic classes, including carbapenems. Other bacteria with high resistance potential included *Pantoea* spp*.* (12 drug classes), *Mangrovibacter* spp*.* (11 drug classes), and the putative pathogen *A. sobria* (7 drug classes). Mapping of metagenomic reads back to MAGs showed an average of ~21.6% of reads mapped, indicating that ARG-host assignments based on MAGs represent only a portion of the community. We also observed uneven MAG recovery between samples, likely reflecting differences in community complexity and bacterial abundance across samples. The agricultural sludge sample showed the lowest mapping overall (~96% unmapped), suggesting that much of the community remains uncaptured. By contrast, MAGs from the animal scats showed higher mapping of metagenomic reads (~43%), but still only captured a portion of the community.

### Isolates with intermediate resistance phenotypes are present in almost all sample sites

**Fig 4:**
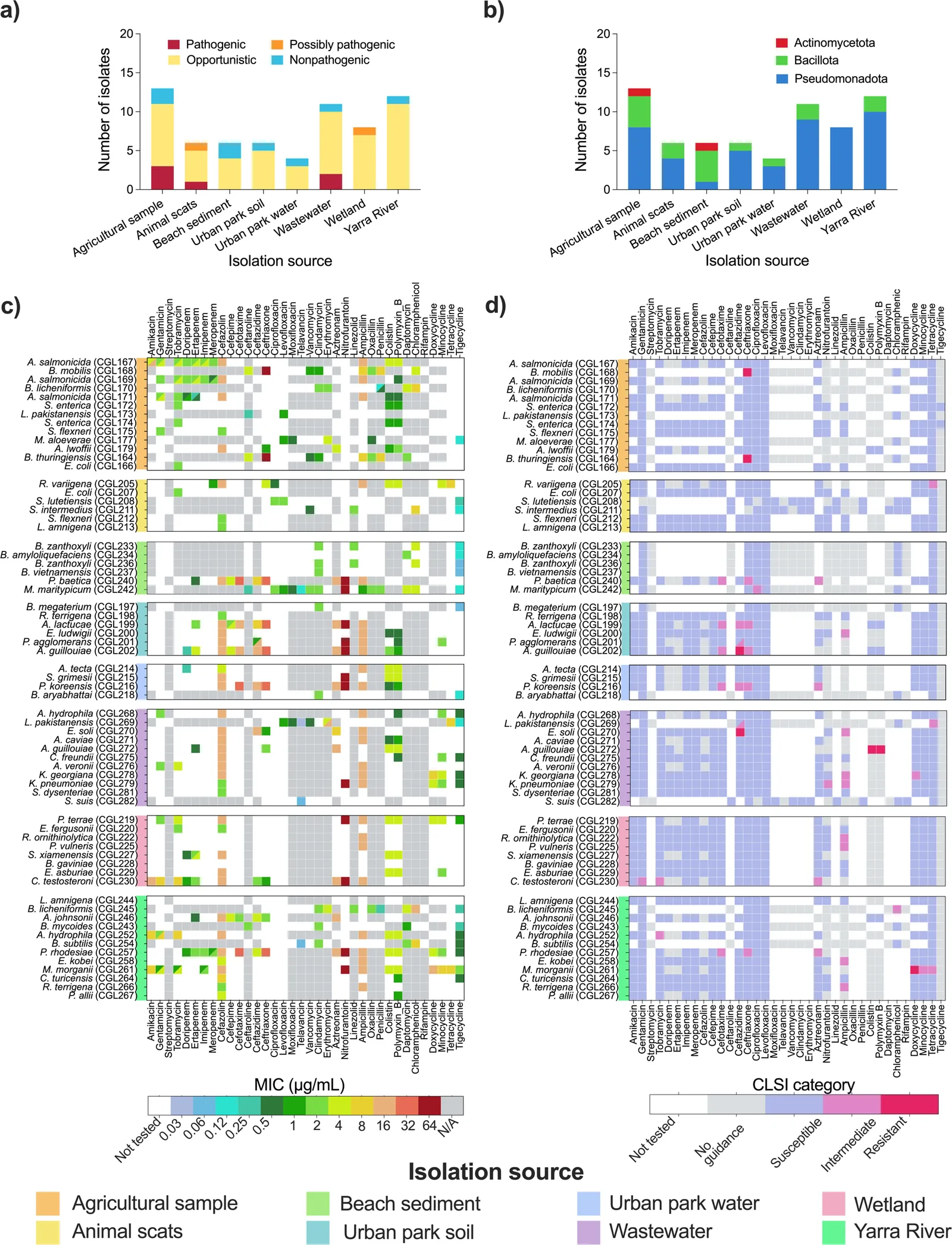
Resistance profiles of isolates by minimum inhibitory concentration (MIC) and as interpreted using Clinical and Laboratory Standards Institute (CLSI, 2018) thresholds. Isolates of sufficient quality (see Materials and Methods) are categorized by (**a**) pathogenicity and (**b**) phylum. (**c**) MIC for each isolate grouped by sampling site. (**d**) Resistance of each isolate as determined by the CLSI guidelines. Cases in which resistance is ambiguous are depicted as cells split along the diagonal, with upper left and lower right indicating the lowest and highest resistance level observed, respectively. Agricultural samples are reported as combined for this analysis.

To support our culture-independent investigation, we selected bacterial isolates from each source for antibiotic susceptibility testing and genomics analysis, with the aim of determining whether AMR genotype is concordant with phenotype, with a particular focus on putative pathogens. Following collection and dilution, samples were plated onto selective and differential media (see Materials and Methods) without antibiotic selection to isolate putative pathogens and other environmental organisms, before subsequent identification with 16S rRNA gene amplicon sequencing. Following culturing, quality control, and presumptive identification through standardized methods, a total of 66 isolates were identified for downstream analysis (Table S9). All samples contained putative pathogens (Fig. 4a), suggesting they are widely distributed across the target environments. Isolates predominantly belonged to Pseudomonadota, with some also from Actinobacteria and Firmicutes (Fig. 4b). Few isolates, including *Escherichia coli* and *Acinetobacter pseudolwoffii,* were represented in the MAGs (Tables S6 and S9). This finding reflects that MAG construction only captured a subset (average of 21.6% metagenome reads mapping to MAGs; Table S5) of the community and is biased toward abundant bacteria, whereas culture-based approaches select primarily for fast-growing or prolific organisms such as pathogens, including those in very low abundance.

To determine the resistance profile of each environmental isolate, MICs for a range of antibiotics were determined, where antibiotics were tested based on isolate Gram status (Fig. 4c). Nine isolates (13.6%) were resistant to antibiotics based on the CLSI guidelines using the most appropriate threshold based on the taxonomy of each organism, although for many taxa, guidance was not available for many antibiotics, and therefore resistance could not be determined (Fig. 4d). For example, CLSI does not currently include specific guidelines for tigecycline, underscoring the need for a better understanding of antibiotic resistance in environmental contexts. All samples indicated at least one isolate showing an intermediate resistance (also known clinically as “susceptible, increased exposure”) phenotype, suggesting that these environments could potentially act as reservoirs for emerging resistance. The number of isolates with intermediate to high resistance according to the CLSI 2018 differed between samples, ranging from 15.4% (agricultural site) to 67.7% (urban park soil). Numbers are affected by sampling bias, as well as CLSI guidance availability for isolated organisms. The isolate with resistance to the highest number of antibiotics was *Morganella morganii* (CGL261), which was resistant to seven different antibiotic classes, including the third-generation antibiotic imipenem (Fig. 4c). Two other opportunistic pathogens, *Citrobacter freundii* (CGL275) (37) and *Proteus tarrae* (CGL219) (38, 39), were each resistant to four antibiotics. While these species are of concern due to their potential pathogenicity to humans, in most cases, resistance was conferred by chromosomal genes.

**Fig 5:**
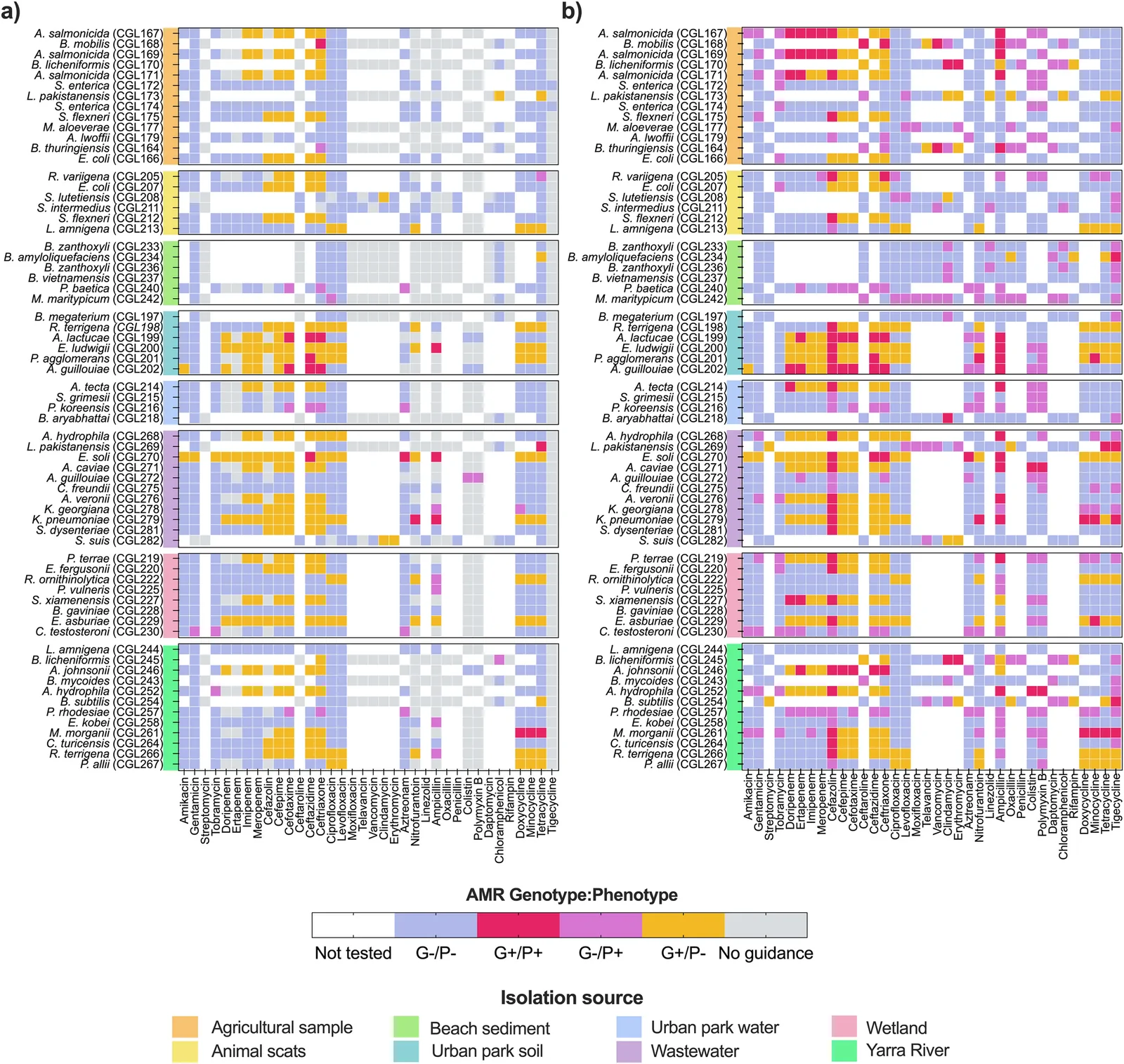
Genotype-to-phenotype contingency plot for antibiotic resistance in environmental isolates. Sampling sites are shown on the left axis of the figure. (**a**) CLSI (2018) thresholds are used to interpret resistance. Resistance here includes intermediate resistance and full resistance as defined by the CLSI (2018). (**b**) Resistance is defined as any MIC above 0 μg/mL to include low-level resistance. Agricultural samples are reported as combined for this analysis.

### Isolate AMR genotype and phenotype are often discordant

To examine whether the AMR phenotypes recorded for each isolate were in accordance with ARG carriage, WGS was conducted for each isolate, and the CLSI and MIC data were overlaid. Considering the CLSI guidelines for resistance, nine isolates showed evidence of agreement between their resistance phenotype and the presence of a corresponding ARG. All isolates showed evidence of susceptible phenotypes when ARGs were not present, as expected (Fig. 5a). However, many isolates also showed susceptible phenotypes, despite carrying an ARG that is predicted to confer resistance to the corresponding antibiotic class. Additionally, several isolates showed a resistant phenotype with no evidence of cognate ARG carriage, suggesting that these isolates either encode uncharacterized resistance genes or have intrinsic antibiotic tolerance mediated by a non-genetic factor. As the CLSI guidelines are not suitable for all bacterial isolates, we also examined resistance using MIC data, where we considered an MIC over 0.1 μg/mL as putative low-level resistance phenotypes (Fig. 5b). Taking MIC into consideration, many of the isolate-antibiotic combinations that previously were either not represented by the CLSI guidelines or showed a susceptible/ARG present phenotype were somewhat resistant. Additionally, the MIC data also indicated more strains had low levels of resistance despite not encoding an ARG; the assumption that an MIC of >0 µg/mL indicates resistance likely increases reporting of false positives, although these isolates could also be evidence of emerging AMR or antibiotic tolerance. ARGs were identified for each isolate (Table S10), where the most common class of ARG detected in these isolates was those conferring resistance to cephalosporins (34.6%) and beta-lactams (25.6%). However, most of the isolates carrying genes conferring this antimicrobial resistance were in fact susceptible to these antibiotics when assayed. There was some overlap in the genes found in isolates to those identified in qPCR array and metagenome screening; for example, *mcr-7.1*, a gene associated with putative colistin resistance, was identified in an *Aeromonas hydrophila* isolate (CGL252) and was also identified in wastewater, beach, agricultural runoff, and urban water metagenomics data sets. However, MIC testing showed this *A. hydrophila* isolate was not resistant to colistin, further highlighting discordance between phenotype and genotype (Tables S3 and S10; Fig. 4). Additionally, while extended spectrum beta-lactamase genes were identified in a number of isolates using a qPCR array (*bla_DHA_*, *bla_VEB_*, and *bla_CTX-1_*), these were less prevalent in metagenomics data sets. *bla_VEB-1_* identified in *Enterobacter soli* (CGL270) was exclusively found in wastewater metagenome samples, and *bla_CTX-M-152_* and *bla_DHA-16_*, which were found in *Kluyvera georgiana* (CGL278) and *Morganella morganii* (CGL261) isolates, respectively, were barely detectable in metagenome samples (1–2 hits in wastewater) (Tables S3 and S10).

A critical issue to address was the extent to which the ARGs are plasmid-associated, given the ready transmission of phenotypes via plasmids. The data showed that most ARGs were chromosomally encoded based on available evidence, suggesting limited capacity for dissemination. Although these chromosomal genes could be associated with other mobile genetic elements such as transposons (Fig. S2). All resistance genes conferring intrinsic resistance were located on the chromosome, while genes associated with acquired resistance were encoded both on plasmids and the chromosome. Only 12.1% of isolates carried ARGs on plasmids, notably *Aeromonas caviae*, *A. hydrophila*, *Lysinibacillus pakistanensis*, *E. soli*, *K. georgiana*, *Acinetobacter johnsonii*, and *Rahnella variigena* isolates, and several plasmids were potentially mobilizable (have *mob* or relaxase genes), indicating they have the potential to be transferred by co-resident conjugative plasmids (Fig. S2).

**Fig 6:**
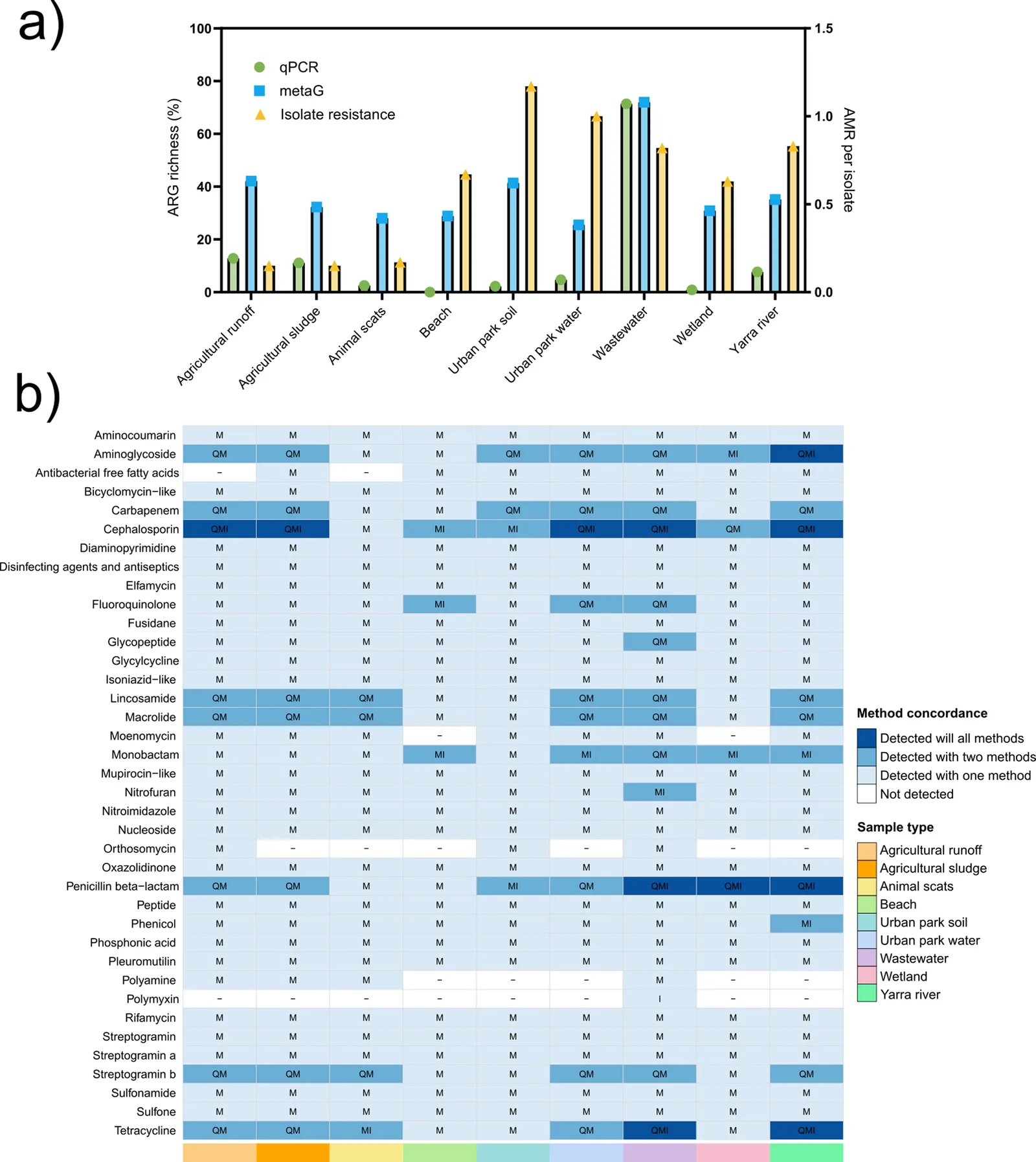
ARG richness, resistance per isolate, and drug class concordance between methods. (**a**) Left y-axis indicates ARG richness for qPCR and metagenomics for each sample type. ARG richness was determined by enumerating unique ARGs per sample and normalized by the total number of detectable ARGs. The right *y*-axis indicates AMR per isolate, which was determined by enumerating intermediate and resistance by CLSI standards for each sample type and normalizing by the number of isolates tested in each sample. (**b**) Drug class concordance between qPCR, metagenomics, and cultured isolates for each sample type is illustrated by a gradient heatmap, where dark blue indicates full concordance for the specified drug class in the specified sample type, middle blue indicates detection by two methods, lightest blue indicates detection by one method, and white indicates not detected by any method. The letters in each tile indicate which methods were concordant, where Q denotes qPCR, M denotes metagenomics, and I denotes isolates.

To further explore concordance between surveillance approaches, we performed a semi-quantitative summary of ARG richness, resistance profiles of cultured isolates, and drug class presence/absence across qPCR, metagenomics, and culture-based methods (Fig. 6). Metagenomics detected greater ARG richness compared to qPCR or cultured isolates, reflecting both its broader detection capacity (>6,000 ARGs) and the potential inflation of ARG richness through detection of genes associated with background ecological processes, including efflux pumps and regulatory functions (Fig. 6a). Although these methods are disparate, which precludes direct statistical comparison, ARG richness showed broad concordance between qPCR and metagenomics, where wastewater showed the greatest ARG richness across both methods (Fig. 6a). This finding suggests that for wastewater which contains human-associated and pathogenic taxa, targeted qPCR can capture ARG diversity comparable to that observed using metagenomics. By contrast, resistance profiles of cultured isolates showed less concordance with qPCR or metagenomics data, with resistance per isolate highest in urban park soil and water when compared to other samples. These results likely reflect that targeted isolation captures a subset of the entire bacterial community.

Drug class profiles were less concordant between methods for each sample (Fig. 6b). Metagenomics detected the greatest number of drug classes overall, consistent with its broader detection capacity and greater number of reference genes in the CARD database compared to targeted qPCR or antibiotics used to test cultured isolates. For the drug classes that qPCR was able to detect, qPCR and metagenomics often detected the same drug classes across samples. Similarly, cultured-based profiles were limited by the antibiotic classes tested in the Sensititre assays. Penicillin beta-lactams, tetracyclines, and cephalosporins showed the greatest concordance across approaches, with putative resistance detected by all three methods in multiple sample types. Complete concordance between methods was observed for cephalosporins in agricultural samples and urban park water; cephalosporins, tetracyclines, and penicillin beta-lactams in wastewater; penicillin beta-lactams in wetland samples; and aminoglycosides, cephalosporins, penicillin beta-lactams, and tetracyclines in Yarra River samples.

## DISCUSSION

This study demonstrates that approaches typically applied in clinical AMR surveillance can be successfully extended to environmental and agricultural contexts. By deploying a coordinated workflow encompassing qPCR, metagenomics, and culture-based genomics across diverse reservoirs, we demonstrate that unified methodologies can profile AMR across the One Health spectrum. This approach contrasts with conventional environmental surveillance, which has often relied on indicator organisms or single-method approaches that underestimate both the diversity and distribution of resistance determinants (4, 24). Clinically aligned methods can be adapted to capture resistance dynamics across environmental compartments, providing a practical model for future One Health surveillance.

We recommend applying multiple methodological pillars for surveillance, as their complementary strengths are required to paint a more complete picture of the environmental resistome. qPCR arrays delivered rapid and sensitive quantification of clinically relevant ARGs, but still provided a narrow perspective (even with 78 genes quantified) and minimal contextual information (e.g., which microbes carry ARGs). Metagenomics broadened this view, revealing a wider spectrum of ARGs, including many harbored by non-pathogenic taxa that would have been overlooked by targeted methods. Many of these ARGs overlap with clinically important resistance genes, mirroring other studies that show environmental and agricultural-derived ARGs detected by metagenomics and metatranscriptomics overlap extensively with clinically important antibiotic resistance genes (36). Many of the highly abundant ARGs identified were resistance-associated rather than explicitly conferring resistance. These findings further highlight that ARG abundance on its own should not be interpreted as active AMR risk. Co-assembly and binning of our data produced over 1,000 species-level MAGs, capturing abundant autochthonous taxa while also identifying putative opportunistic pathogens. Despite this depth, MAGs provided only a partial perspective on low-abundance pathogens and mobile genetic elements; additionally, DNA-based methods detect extracellular ARGs, highlighting the continued importance of more targeted approaches, including culture-based phenotyping. Additionally, mapping reads back to MAGs showed an average of 21.6%, suggesting that only a portion of the community was captured using this method, and therefore only a proportion of ARG-host assignments are reflected in these data. While MAGs provide valuable information regarding the potential hosts and genomic context of resistance determinants, they cannot capture ARGs associated with organisms that were not sufficiently abundant to be successfully assembled into MAGs. The traditional approach of culture-based isolation remains best practice for functional validation and allows genotype-to-phenotype links to be tested, though this is biased to only a subset of organisms and excludes the environmental majority that are recalcitrant to culturing, which was further highlighted by the low mapping rate observed when aligning metagenome reads to isolate genomes.

Combining these approaches also highlighted discrepancies between predicted and observed resistance, including intermediate phenotypes. In some cases, ARGs were detected without corresponding resistance, likely reflecting differences in gene expression, reading frame, copy number, or genomic background (40). Conversely, resistance without identifiable genetic determinants was also observed, potentially pointing to the contribution of intrinsic mechanisms to antibiotic tolerance or the existence of novel or uncharacterized resistance genes. Examples of this include non-carbapenemase-dependent carbapenem-resistant phenotypes in *Klebsiella* that depend on changes in the outer membrane permeability to provide intrinsic resistance to carbapenems (41). Together, these findings underscore that carriage of ARGs does not always equate to phenotypic resistance and that phenotype can emerge independently of known genotypes.

The CLSI guidelines (CLSI, 2018) provide a clinically relevant accounting of resistance for applicable species, which remain indispensable for patient management and clinical surveillance. However, their application to environmental and non-clinical taxa is less straightforward. The behavior of a resistance gene can vary considerably depending on its mode of transmission, copy number, and the genomic background of the host, meaning that CLSI interpretive breakpoints may not be useful predictors of emerging resistance. At the same time, persistence of ARGs without conferring phenotypic resistance is possible and may obscure the expected link between genotype and phenotype (42). This is why well-controlled MIC testing remains essential for accurately characterizing phenotypic resistance in environmental isolates. Nevertheless, persistence of “silent” ARGs can still pave the way for the emergence of resistance (40). Together, these factors highlight both the value and the limitations of applying clinical interpretive frameworks to environmental resistance data and underscore the need for parallel ecological and functional perspectives.

Currently, there are no equivalent frameworks for interpreting environmental resistomes. Developing an effective framework for understanding AMR risk requires a nuanced approach that accounts for factors such as the host, ARG mobility, local selective pressures, and the co-occurrence of primary or opportunistic pathogens that could receive ARGs from environmental hosts. Without these contextual factors, it remains difficult to assess whether a given resistome profile represents a benign feature of the environment or an emerging risk. Several studies have taken steps to incorporate these components into their surveillance strategies by measuring selective agents, including antibiotic residues and selective factors such as heavy metals or biocides, and integrating these data with paired metagenomic analysis (43, 44). Our combined approach helps to expose some of these weaknesses, demonstrating how much interpretative power is lost when surveillance depends upon a single method or perspective. This contributes to the broader goal of building an interpretive framework for surveillance of environmental AMR that is tailored to complex sample types and microbial communities.

We acknowledge that our study has several limitations that highlight the need for cautious interpretation of environmental AMR. Assembly of repetitive or mosaic sequences using short-read metagenomic data is challenging and can hamper the detection of plasmids, which are an integral vehicle for the carriage and transmission of ARGs. In an attempt to capture the fraction of the resistome that was not reflected in our MAGs, we performed ARG screening on unassembled metagenome reads, although this approach does not resolve ARG context (host or genomic linkage) and does not provide information about gene mobility. Several studies have begun incorporating methods such as Hi-C or long-read sequencing to capture MGE-associated ARGs, and future work should prioritize this (45, 46). Additionally, our study relied primarily on DNA-based methods; therefore, we likely captured extracellular ARGs and ARGs from non-viable organisms, which may have artificially inflated AMR estimates. Furthermore, our study utilized non-selective agar for the agnostic isolation of putative pathogens from the environment, which likely biased toward fast-growing, aerobic, non-resistant organisms. In future, a balanced approach wherein selective and non-selective agar is used for isolation could be implemented. Future studies should also seek to integrate measurements of environmental selective pressures (antibiotics, metals, or biocides) to help contextualize resistome findings. Finally, our study shows a snapshot of these environments at the time of sampling; future longitudinal studies with increased sample sizes and sites will provide more data for statistical interpretation and allow greater generalization across environments.

While the comprehensive multi-method workflow applied here would be difficult to implement routinely across large public health surveillance programs, it provides a benchmark for understanding the strengths and limitations of individual approaches. qPCR offers a rapid and cost-effective option for targeted monitoring of priority resistance genes, metagenomics provides broader resistome surveillance and discovery capacity, and culture-based genomics remains essential for linking genotypes to phenotypes. The relative value of these approaches will depend on whether surveillance programs are focused on tracking a defined set of high-risk resistance genes across numerous samples or on obtaining deeper insights into resistome diversity, emerging resistance mechanisms, and transmission pathways. Considerations such as cost, technical capacity, sample throughput, and reporting timelines will also influence method selection. Our findings therefore support an adaptive One Health approach in which surveillance strategies are tailored to both the questions being asked and the resources available. Importantly, the methods evaluated here are readily transferable across environmental, agricultural, and clinical samples, demonstrating that common surveillance approaches can generate comparable and complementary data sets across traditionally siloed sectors and thereby support more integrated surveillance frameworks.

Overall, we observed a generally low prevalence outside of wastewater-associated samples but high diversity of resistance determinants. This finding likely reflects both the relatively high-income setting of Melbourne (established waste management protocols to reduce bacterial load, etc.). Wastewater was a critical reservoir, containing an array of clinically important determinants, reinforcing the value of wastewater epidemiology (47). Beyond known pathogens, ARGs were frequently identified in endemic species such as those of *Pantoea* and *Mangrovibacter*, many of which carried genes spanning multiple antibiotic classes. This result reinforces previous observations that environmental bacteria can act as reservoirs of resistance, even when not pathogenic, highlighting their potential role in horizontal gene transfer to clinically relevant species (48). These results provide additional high-resolution evidence of ARG carriage in non-pathogenic hosts across both urban and agricultural environments, demonstrating that even in a relatively low-prevalence setting, diverse resistance mechanisms occur outside clinical settings. Future work could now extend this framework across broader geographic and socioeconomic contexts and through longitudinal monitoring to better resolve how resistomes evolve under different selective pressures.

## MATERIALS AND METHODS

### Collection of samples

Sample collection, processing, and analysis pipelines are outlined in Fig. S3. Samples were collected (Table S1) in sterile 50-mL centrifuge tubes in triplicate and processed within 48 h. For bacterial isolation, soil and sand were processed by resuspending 1 g in 9 mL of sterile peptone saline diluent (1 g/L peptone, 8.5 g/L NaCl, pH 7.0), and plating 10 µL of the supernatant onto three differential and/or selective agar plates: sheep blood agar (SBA) (for differential identification of hemolytic bacteria), bile esculin agar (BEA) (for *Enterococci*), and MacConkey agar (MAC) (for gram-negative fecal/enteric bacteria) plates (Media Preparation Unit, Department of Microbiology & Immunology, The University of Melbourne). Water samples were directly plated or serially diluted and directly plated onto SBA, BEA, and MAC. Plates were incubated aerobically at 37°C for 24 h. Several colonies with different morphologies and biochemical activity indicated by selective plates were chosen and re-streaked onto a fresh selective agar plate and incubated at 37°C for 24–48 h to further isolate the strain. A single colony from each selective agar plate was re-streaked onto a Luria-Bertani agar plate (10 g/L tryptone, 5 g/L yeast extract, 5 g/L NaCl, and 15 g/L agar) and incubated at 37°C for 24–48 h. For long-term storage, bacterial stocks were prepared by culturing isolates in 10 mL LB broth (10 g/L tryptone, 5 g/L yeast extract, and 5 g/L NaCl) at 37°C with shaking at 200 rpm for 24–48 h and stored in 25% (vol/vol) sterile glycerol at −80°C.

### Bacterial isolate identification

Bacterial isolates were identified based on colony morphology and biochemical reactions on the selective agar plates as well as Sanger sequencing of the 16S rRNA gene. Single colonies on the LB agar plates were picked for colony PCR by resuspending the cells in 100 µL of UltraPure DNase/RNase-Free Distilled Water (Thermo Fisher Scientific) and boiling at 100°C for 10 min. The tubes were cooled on ice for 10 min before centrifugation at 16,000 × *g* for 10 min. The 16S rRNA gene was amplified from the supernatant using universal 16S rRNA gene primers (27F (5′-AGAGTTTGATCCTGGCTCAG-3′) and 1492R (5′-GGTTACCTTGTTACGACTT-3′), with an expected amplicon length of ~1,400 bp. Individual reactions contained 1× Taq MasterMix (New England Biolabs), 400 µM of each primer, and 2 µL of the DNA template, mixed to a final volume of 50 µL with UltraPure DNase/RNase-Free Distilled Water. Cycling conditions for PCR amplification were as follows: 95°C for 2 min followed by 35 cycles of 95°C for 30 s, 56°C for 30 s, 68°C for 1 min, with the final cycle ending at 68°C for 5 min before cooling down to 4°C. The PCR products were loaded on a 1% wt/vol agarose gel at 90V for 35 min. The gel was visualized with a UV transilluminator, and the band of the expected size (~1,400 bp) was cut and extracted using the ISOLATE II PCR and Gel Kit (Bioline) before sending for Sanger sequencing at Micromon Genomics (Monash University). If DNA was not amplifiable, genomic DNA was extracted as described above and used as a template for 16S rRNA gene PCR. The sequences were compared against the Standard Nucleotide BLAST database (NCBI) using a percent identity threshold of 97%.

### Isolate genomic DNA extraction

gDNA was extracted using a CTAB method as follows: 2–4 mL of cell culture was pelleted by centrifugation at 11,000 × *g,* and the supernatant was discarded, followed by 30 μL of lysozyme solution (50 mg/mL in Tris-HCl, pH 8), followed by vortexing and incubation at 37°C for 1 h. Following incubation, 70 μL of 10% SDS and 10 μL of 20 mg/mL proteinase K were added and incubated for 15 min at 65°C. Next, 100 μL of 5 M NaCl and 100 μL of CTAB solution (10% CTAB in 0.7 M NaCl) were added, vortexed, and incubated for 10 min at 65°C. Next, 750 μL of chloroform was added, followed by gentle mixing and centrifugation at 11,000 × *g* for 15 min. The aqueous phase was transferred to a fresh tube and precipitated using 0.6 volumes of isopropanol and incubated at −20°C for 30 min. The precipitate was collected via centrifugation at 13,000 × *g* for 10 min before washing with 1 mL of ice-cold 75% ethanol, and final centrifugation at 13,000 × *g* for 5 min prior to air drying for 30 min. The DNA pellet was resuspended in 50 μL of UltraPure water and left at 4°C to ensure dissolution.

### Antimicrobial susceptibility test

Sixty-six isolates were selected for antimicrobial susceptibility testing (AST) using Sensititre plates (Thermo Fisher). Gram-negative strains were evaluated using Sensititre Gram-Negative GN6F and Sensititre Gram-Negative GNX2F plates. Gram-positive strains were analyzed on Sensititre Gram-Positive GPALL3F plates (Sensititre Plate Guide Booklet). The cultures were streaked from glycerol stocks onto LB agar plates and incubated overnight at 37°C. Colonies were selected from each plate and resuspended in phosphate-buffered saline solution (13.69 mM NaCl, 2.68 mM KCl, 10.14 mM Na_2_HPO_4_, 1.76 mM KH_2_PO_4_, pH 7.4) and adjusted to a turbidity equivalent to 0.5 McFarland standard (0.24 mM BaCl_2_ · 2H_2_O in 1% [vol/vol] H_2_SO_4_) or OD_600_ ≈ 0.15–0.18 and transferred 1–30 µL to Mueller-Hinton broth (Oxoid, Thermo Fisher). Following adjustment, 50 µL of the culture was loaded into each well of the Sensititre plate, sealed with perforated sealing labels, and incubated at 37°C for 18–24 h. Interpretation of Sensititre results was performed in accordance with the CLSI & Antimicrobial Susceptibility Testing M100 guidelines.

### qPCR

QIAGEN microbial DNA qPCR arrays for Antibiotic Resistance Genes were used to screen environmental samples for 78 ARGs as per the manufacturer’s instructions. All qPCR preparation was conducted inside a UV-decontaminated class II biosafety cabinet. Briefly, positive control DNA obtained from QIAGEN was diluted 1:10 before mixing with qPCR Mastermix, where 40.8 µL of positive control DNA was mixed with 510 µL of Mastermix and 469.2 µL of PCR-grade H_2_O. Sample gDNA was diluted to a concentration of ~200 ng and added to qPCR Mastermix and PCR-grade water as described for the positive control. Each mixture was poured into a reservoir before loading 10 µL into each well on the 384-well plate. The assay card was tightly sealed with the optical adhesive and centrifuged at 1,500 × *g* for 1 min. Plates were subsequently loaded into the qPCR thermocycler (QuantStudio 7 Flex, Applied Biosystems) with cycling conditions as follows: initial denaturation 1 cycle 10 min at 95°C, followed by 40 cycles of denaturation 15 s at 95°C and 2 min annealing extension at 60°C. qPCR results were quality controlled in QuantStudio Real-Time PCR software v1.3, and data analysis was conducted using the analysis spreadsheet provided by QIAGEN.

### Whole-genome sequencing of isolates

Genomic DNA from each of the 66 isolates was extracted as described above and sent for whole-genome sequencing (the MHTP Medical Genomics Facility, Hudson Institute). Libraries were prepared using the Celero EZ DNA-Seq library prep kit. Paired-end short-read (150 bp) sequencing was performed with the Illumina NextSeq2000 platform. Quality control of the short-read libraries was evaluated using FastQC v0.11.7 (49), and adapters were trimmed from the reads using FastX ToolKit v0.0.13 (https://github.com/agordon/fastx_toolkit). Genomes were assembled with Unicycler v0.4.7 (50), and CheckM2 v1.1.0 (51) and QUAST v5.0.2 (52) were used to evaluate the quality of the assemblies (Table S7). Genome Taxonomy Database (GTDB)-Tk v2.32 was used to assign assemblies to the GTDB (r214) taxonomy (53, 54). Isolates were removed from the analysis if their GTDB-assigned taxonomy differed from 16S rRNA gene PCR identification above the rank of genus, or if they could not be classified by GTDB-Tk, likely due to contamination. A final set of 66 isolates was maintained for downstream analysis and had congruent taxonomy assignments at the rank of genus (Table S9).

### Plasmid and antibiotic resistance gene identification

Plasmids were predicted using PlasClass 06.2020 (55) with a cutoff of 0.7 to classify contigs from draft assemblies, as well as the function MOB-recon from MOB-Suite (56). Contigs for which PlasClass and MOB-Recon results were incongruent were manually examined and aligned with NCBI BLAST to the non-redundant nucleotide database to filter out contigs likely to be chromosomal. Prodigal v2.6.3 (57), implemented in Assembly2gene (https://github.com/LPerlaza/Assembly2Gene), was used to identify ARGs (Table S10). Then the predicted genes were aligned with NCBI BLAST against a curated non-redundant ARGs database containing 6,657 genes, combining AMRfinderPlus (58), the CARD (59), ResFinder (60), Beta-Lactamase DataBase (61), and membrane components such as porins and efflux pumps. Assembly2gene generates gene alignments and predicted peptides using R packages “msa,” “reshape2,” “Biostrings,” and “seqinr.” Heatmaps and plots were generated using R packages “ggplot2” and PRISM (v9.0.0; Apple MAC, GraphPad Software, San Diego, California, USA, https://www.graphpad.com). Putative ARG resistance mechanisms as provided by the CARD were compared with the MIC data to link isolate genotypes to their resistance profiles.

### Metagenomics

Prior to DNA extraction, samples were subjected to one round of bead-beating with FastPrep-24 (MP Biomedicals) (6.5 m/s for 30 s); standard sample amount and reagent volumes were used as per the manufacturer’s instructions. DNA was extracted from samples (Table S11) using the QIAGEN DNeasy PowerSoil kit, as per the manufacturer’s instructions, with an elution volume of 30 μL. Samples were sequenced at the Australian Centre for Ecogenomics, using an Illumina NextSeq2000, to generate 2 × 150 bp paired-end reads (average of ~120 million paired reads per sample). Reads were assessed for quality and trimmed using fastp (62), which included the removal of adapters and low-quality sequences, as well as filtering by read length (≥50 bp) and average quality score (≥30), with automatic adapter detection enabled. To enhance genome recovery, particularly of low-abundance taxa, reads from within each environment were co-assembled using MEGAHIT v1.2.9 with default parameters (63). Assemblies were filtered to retain contigs ≥1,000 bp. Genome binning was independently performed with four algorithms: Vamb v4.1.3 (64), MetaBAT v2.12.1 (65), CONCOCT v1.1.0 (66), and SemiBin2 (67). Bins were refined with the bin refinement module of MetaWRAP (68). Bins were dereplicated with dRep v3.4.244 (69) at a 95% ANI threshold corresponding to the species level, incorporating CheckM2 metrics. Completeness and contamination were assessed with CheckM2 (51). In total, we recovered 1,032 bacterial MAGs meeting the MIMAG criteria for medium quality (≥50% completeness; <10% contamination) and high quality (≥90% completeness; <5% contamination). MAGs were taxonomically classified using GTDB-Tk v2.3.2 against the Genome Taxonomy Database release R214 (53). A maximum-likelihood phylogenetic tree of bacterial MAGs was built using GTDB-Tk v2.3.2 with the hmmalign 3.4 model, then visualized using iTOL v6 (70) and edited in Inkscape. MAGs for each metagenomic sample were screened for ARGs using RGI. Coverage and relative abundance of each MAG in each data set were determined with coverM (71). In addition, each metagenome short-read data set was separated into multiple 20 million read subsets (e.g., 100 million reads split into 5 × 20 million read subsets) using seqtk (v1.5) (https://github.com/lh3/seqtk), and each subset was screened for ARGs using RGI before the counts were consolidated. Consolidated counts for each data set were used to calculate relative abundance based on the number of reads mapped to ARG class divided by the total number of reads for each data set, and the number of reads in each data set are listed in Table S12.

## Supplementary Material

Reviewer comments

## Data Availability

All raw metagenome and isolate whole-genome sequencing data generated in this study are deposited in the NCBI Sequence Read Archive (SRA) under the BioProject accession number PRJNA1514836.

## SUPPLEMENTAL MATERIAL

Supplemental material msystems.00741-26-s0001.docx Supplemental table legends; Fig. S1 to S3.

Supplemental tables msystems.00741-26-s0002.xlsx Tables S1 to S12.

OPEN PEER REVIEW reviewer-comments.pdf An accounting of the reviewer comments and feedback.

ASM does not own the copyrights to Supplemental Material that may be linked to, or accessed through, an article. The authors have granted ASM a non-exclusive, world-wide license to publish the Supplemental Material files. Please contact the corresponding author directly for reuse.
