# Supplementary material for: Integrated cross-sectoral surveillance of antimicrobial resistance genotypes and phenotypes across disparate reservoirs
Source: mSystems. 2026 Sep 28;11(10):e00741-26. doi: 10.1128/msystems.00741-26 (PMC13712752; doi:10.1128/msystems.00741-26)
Supplement: Supplementary file 1 [file reviewer-comments.pdf]

ASM | mSystems

Integrated cross-sectoral surveillance of antimicrobial resistance genotypes and phenotypes across disparate reservoirs

Thomas Watts, Thanavit Jirapanjawat, Laura Perlaza-Jiménez, Francesco Ricci, Eve Tudor-Matthew, Eleonora Chiri, Sean Bay, Rhys Grinter, Rachael Lappan, Trevor Lithgow, Laura Woods, and Chris Greening

Corresponding Author(s): Thomas Watts, Monash University

Review Timeline:

Submission Date:	June 10, 2026
Editorial Decision:	July 16, 2026
Revision Received:	August 23, 2026
Accepted:	September 3, 2026

Editor: Erica Hartmann

Reviewer(s): Disclosure of reviewer identity is with reference to reviewer comments included in decision letter(s). The following individuals involved in review of your submission have agreed to reveal their identity: Ishi Keenum (Reviewer #1); Rahat Zaheer (Reviewer #2)

Transaction Report:

(Note: With the exception of the correction of typographical or spelling errors that could be a source of ambiguity, letters and reports are not edited. The original formatting of letters and referee reports may not be reflected in this compilation.)

DOI: https://doi.org/10.1128/msystems.00741-26

Re: mSystems00741-26 (**Integrated cross-sectoral surveillance of antimicrobial resistance genotypes and phenotypes across disparate reservoirs**)

Dear Prof. Chris Greening:

Thank you for the privilege of reviewing your work. Below you will find my comments, instructions from the mSystems editorial office, and the reviewer comments.

Please return the manuscript within 60 days; if you cannot complete the modification within this time period, please contact me. If you do not wish to modify the manuscript and prefer to submit it to another journal, notify me immediately so that the manuscript may be formally withdrawn from consideration by mSystems.

Revision Guidelines
To submit your modified manuscript, log into the submission site at https://msystems.msubmit.net/cgi-bin/main.plex. Go to Author Tasks and click the appropriate manuscript title to begin. The information you entered when you first submitted the paper will be displayed; update this as necessary. Note the following requirements:

• Upload point-by-point responses to the issues raised by the reviewers in a file named "Response to Reviewers," NOT in your cover letter.
• Upload a compare copy of the manuscript (without figures) as a "Marked-Up Manuscript" file.
• Upload a clean .DOC/.DOCX version of the revised manuscript and remove the previous version.
• Each figure must be uploaded as a separate, editable, high-resolution file (TIFF or EPS preferred), and any multipanel figures must be assembled into one file.
• Any supplemental material intended for posting by ASM should be uploaded with their legends separate from the main manuscript. You can combine all supplemental material into one file (preferred) or split it into a maximum of 10 files with all associated legends included.

For complete guidelines on revision requirements, see our Submission and Review Process webpage. Submission of a paper that does not conform to guidelines may delay acceptance of your manuscript.

Data availability: ASM policy requires that data be available to the public upon online posting of the article, so please verify all links to sequence records, if present, and make sure that each number retrieves the full record of the data. If a new accession number is not linked or a link is broken, provide mSystems production staff with the correct URL for the record. If the accession numbers for new data are not publicly accessible before the expected online posting of the article, publication may be delayed; please contact production staff (mSystems@asmusa.org) immediately with the expected release date.

Publication Fees: For information on publication fees and which article types are subject to charges, visit our website. If your manuscript is accepted for publication and any fees apply, you will be contacted separately about payment during the production process; please follow the instructions in that e-mail. Arrangements for payment must be made before your article is published.

ASM Membership: Corresponding authors may join or renew ASM membership to obtain discounts on publication fees. Need to upgrade your membership level? Please contact Customer Service at Service@asmusa.org.

The ASM Journals program strives for constant improvement in our submission and publication process. Please tell us how we can improve your experience by taking this quick Author Survey.

Thank you for submitting your paper to mSystems.

Sincerely,
Erica Hartmann
Editor
mSystems

Reviewer #1 (Comments for the Author):

The authors have addressed my concerns. I appreciate the addition of the limitations and the extensive re-analysis they underwent. This is a strong contribution to the literature and will further our discussions on how to harmonize methodology in environmental AMR surveillance and I hope it will lead to further discussion on how to also develop robust One Health surveillance, given our differing limitations across fields.

Reviewer #2 (Comments for the Author):

This study evaluates a multi-method framework for antimicrobial resistance (AMR) surveillance across diverse environmental and agricultural reservoirs. By integrating qPCR arrays, metagenomics, metagenome-assembled genomes (MAGs), culture-based isolation, antimicrobial susceptibility testing, and whole-genome sequencing, the authors compare the strengths and limitations of different approaches for detecting and characterizing AMR across a range of sample types.
The authors have improved the manuscript and addressed most of the concerns raised during the first round of review. The revised version provides a better discussion of methodological limitations, incorporates re-analysis of the metagenomic datasets, and highlights the complementary nature of qPCR, metagenomic, and culture-based surveillance approaches. However, in reviewing the revised manuscript, I identified several additional issues that were not raised previously but warrant further consideration. These comments are outlined below.
The statement that clinically aligned methods could provide a "resolution comparable to hospital-based monitoring" remains a bit strong. Given the limitations such as low MAG recovery (~20% of reads), incomplete resolution of plasmid-associated ARGs, imperfect genotype-phenotype concordance, and challenges applying clinical breakpoints to environmental isolates, the data support the applicability of clinical surveillance tools to environmental samples, but not necessarily equivalent resolution to hospital surveillance systems. I recommend softening this further with limitations stated clearly.
Methods/results: There appear to be inconsistencies in the reported sequencing instruments used throughout the manuscript. In the Results section, metagenomic sequencing is described as being performed on a NextSeq100, whereas the Methods section states that metagenomic libraries were sequenced on a NextSeq5000. In addition, I am not aware of an Illumina platform designated "NextSeq5000," suggesting this may be a typo. I think currently marketed instruments are typically NextSeq 500, NextSeq 550, NextSeq 1000, and NextSeq 2000. The authors should verify and correct the sequencing platform used for metagenomic sequencing and ensure consistent reporting throughout the manuscript. Considering that, please make sure that the NextSeq2000 reported for isolate whole-genome sequencing is correct. Accurate reporting of sequencing instrumentation is important for reproducibility and interpretation of sequencing depth and data quality.
The Methods still state that datasets were subsampled to 20 million reads before RGI analysis, whereas the response to reviewers indicates that the full metagenomic datasets were re-analyzed. Please clarify which approach was used for the final results and update the Methods accordingly. It would also be helpful to report the range of sequencing depths across samples.
The manuscript now includes some examples comparing qPCR, metagenomic, and culture-based findings, which partially addresses my previous concern. I see "A similar distribution of beta-lactamase variant..." added (line 178) and "There was some overlap in the genes found in isolates to those identified in qPCR array and metagenome screening..." (lines 295 onwards). However, the comparison remains largely anecdotal. The original comment (I agree with that comment) by reviewer 1 was aimed at understanding the degree of concordance among surveillance approaches. For example, were patterns of ARG richness and resistance classes consistent across qPCR, metagenomics, and cultured isolates? A brief quantitative summary (even descriptive rather than statistical) of agreement and disagreement among methods would strengthen the manuscript and better support the conclusion that the approaches provide complementary information.
Lines 211-212: Inflation of efflux genes (issue also raised by reviewer #2): The interpretation of fluoroquinolone resistance genes requires additional caution. Most of the genes listed here (adeF, marA, soxS, soxR, sdrM, rsmA, acrA, acrB, acrE, acrF, acrS, emrA, emrB, emrR, mdtH, patB) do not directly confer clinically significant fluoroquinolone resistance in the same way as plasmid-mediated determinants (e.g., qnr genes, aac(6′)-Ib-cr, qepA, oqxAB) or QRDR mutations in gyrA, gyrB, parC, and parE. Rather, many of these genes encode efflux pumps or regulatory systems that contribute to reduced susceptibility and may only result in clinically relevant resistance when combined with other resistance mechanisms.
Several of these efflux genes are core chromosomal components that may be present in fully susceptible strains. In many cases, resistance is associated with altered expression resulting from mutations in regulatory genes (e.g., marR, acrR, soxR, ramR) rather than the mere presence of the genes themselves. Therefore, detection of these genes by CARD/RGI should be interpreted as putative resistance potential rather than evidence of fluoroquinolone resistance. This limitation should be acknowledged more explicitly in the Results and/or Discussion, particularly given the widespread fluoroquinolone-associated signals observed across sample types in Figures 2 and 3. The revised manuscript does acknowledges that many RGI hits represent intrinsic resistance determinants, stress-response systems, or efflux components. However, Figures 2 and 3 still treat all ARG classes similarly.
As mentioned above for fluoroquinolones, aminoglycoside, and disinfectant-associated categories may also be heavily influenced by genes such as regulators and efflux systems. I suggest emphasizing that ARG abundance should not be interpreted directly as AMR risk, particularly when many detected determinants likely represent intrinsic ecological functions.
Table S3: The reported abundance of rpoB2 deserves further clarification. Rifampicin resistance is generally mediated by specific mutations within the RNA polymerase β-subunit (rpoB) rather than by gene presence. The manuscript should clarify whether the detected sequences contained known resistance-conferring mutations or whether annotation was based solely on homology to resistance-associated rpoB alleles. This is particularly important because Table S8 reports a best sequence identity of only ~92%, which may not be sufficient to confidently infer a resistance phenotype.
Table S3: Consider adding a 'Drug Class' column before the 'Gene' column. This would make it easier for readers to interpret the table and relate individual ARGs to the resistance classes discussed throughout the manuscript.
Table S8: Several resistance hits have low sequence identity., e.g., glycopeptide resistance hits, which are also based on individual van-cluster genes (e.g., vanT, vanW, vanY) detected at low sequence identity (~33-40%). Please clarify if complete or near-complete resistance operons were ever identified.

The manuscript notes that an average of only 21.6% of metagenomic reads mapped back to recovered MAGs, with some samples showing up to 96% unmapped reads. While this is not unexpected for complex environmental communities, it highlights that ARG-host assignments derived from MAGs represent only a subset of the total community. It would be helpful if the authors briefly discussed which sample types exhibited the lowest MAG recovery and how this may influence interpretation of MAG-associated ARG profiles.
The reported sequencing depth is inconsistent. The Results indicate ~120 million paired reads per sample (Line 158), whereas the Methods report ~238 million reads per sample (line 574). Please clarify whether these values represent read pairs versus total reads.
Line 427: "Overall, we observed a relatively low prevalence but high diversity of resistance determinants" would benefit from clarification of how "prevalence" is defined. Wastewater samples showed substantial ARG burdens, whereas other environments showed lower abundance. It may be more accurate to specify that prevalence was generally low outside wastewater-associated samples.
Additionally, the limited number of samples/sites per environment restricts the ability to generalize these findings and should be acknowledged when drawing broader conclusions about environmental AMR patterns.

Response to reviewers:

We thank both reviewers for taking the time to revaluate our manuscript and for their helpful comments and insights. Our responses to the reviewer's comments are outlined below:

Reviewer #1 (Comments for the Author):

The authors have addressed my concerns. I appreciate the addition of the limitations and the extensive re-analysis they underwent. This is a strong contribution to the literature and will further our discussions on how to harmonize methodology in environmental AMR surveillance and I hope it will lead to further discussion on how to also develop robust One Health surveillance, given our differing limitations across fields.

We thank the reviewer for taking the time to read our manuscript and for their valuable comments that have improved the study.

Reviewer #2 (Comments for the Author):

This study evaluates a multi-method framework for antimicrobial resistance (AMR) surveillance across diverse environmental and agricultural reservoirs. By integrating qPCR arrays, metagenomics, metagenome-assembled genomes (MAGs), culture-based isolation, antimicrobial susceptibility testing, and whole-genome sequencing, the authors compare the strengths and limitations of different approaches for detecting and characterizing AMR across a range of sample types. The authors have improved the manuscript and addressed most of the concerns raised during the first round of review. The revised version provides a better discussion of methodological limitations, incorporates re-analysis of the metagenomic datasets, and highlights the complementary nature of qPCR, metagenomic, and culture-based surveillance approaches. However, in reviewing the revised manuscript, I identified several additional issues that were not raised previously but warrant further consideration. These comments are outlined below.

The statement that clinically aligned methods could provide a "resolution comparable to hospital-based monitoring" remains a bit strong. Given the limitations such as low MAG recovery (~20% of reads), incomplete resolution of plasmid-associated ARGs, imperfect genotype-phenotype concordance, and challenges applying clinical breakpoints to environmental isolates, the data support the applicability of clinical surveillance tools to environmental samples, but not necessarily equivalent resolution to hospital surveillance systems. I recommend softening this further with limitations stated clearly.

We agree with the reviewer that this comment is a strong assertion given the limitations and outcomes, we have therefore removed the statement 'resolution comparable to hospital-based monitoring' from the text.

Methods/results: There appear to be inconsistencies in the reported sequencing instruments used throughout the manuscript. In the Results section, metagenomic sequencing is described as being performed on a NextSeq100, whereas the Methods section states that metagenomic libraries were sequenced on a NextSeq5000. In addition, I am not aware of an Illumina platform designated "NextSeq5000," suggesting this may be a typo. I think currently marketed instruments are typically NextSeq 500, NextSeq 550, NextSeq 1000, and NextSeq 2000. The authors should verify and correct the sequencing platform used for metagenomic sequencing and ensure consistent reporting throughout the manuscript. Considering that, please make sure that the NextSeq2000 reported for isolate whole-genome sequencing is correct. Accurate reporting of sequencing instrumentation is important for reproducibility and interpretation of sequencing depth and data quality.

We thank the reviewer for highlighting these errors, 'Nextseq5000' and 'Nextseq100' were regrettable typographic errors. WGS and metagenomics sequencing was performed with the 'Nextseq2000' platform.

The Methods still state that datasets were subsampled to 20 million reads before RGI analysis, whereas the response to reviewers indicates that the full metagenomic datasets were re-analyzed. Please clarify which approach was used for the final results and update the Methods accordingly. It would also be helpful to report the range of sequencing depths across samples.

We thank the reviewer for identifying the lack of clarity in the methods. The entire metagenomics datasets for each sample were reanalysed using RGI. We analysed each full dataset by splitting them into 20 million read subsets, e.g. 100 million reads into 5 x 20 million read 'chunks'. This allowed the analysis to be less resource intensive and we could run our analysis in parallel. We consolidated the counts for each subset to provide an output for the full metagenomics dataset. As the reviewer requested, we have provided the range of sequencing depths for each read set reported by seqtk in the supplementary Table S12.

The methods have been updated to improve clarity as follows (line 634-641):

'In addition, each metagenome short reads dataset was separated into multiple 20 million read subsets (e.g. 100 million reads split into 5 x 20 million read subsets) using seqtk (v1.5) (https://github.com/lh3/seqtk) and each subset was screened for ARGs using RGI, before the counts were consolidated. Consolidated counts for each dataset were used to calculate relative abundance based on the number of reads mapped to ARG class divided by total number of reads for each dataset, the number of reads in each dataset are listed in **Table S12**.'

The manuscript now includes some examples comparing qPCR, metagenomic, and culture-based findings, which partially addresses my previous concern. I see "A similar distribution of beta-lactamase variant..." added (line 178) and "There was some overlap in the genes found in isolates to those identified in qPCR array and metagenome screening..." (lines 295 onwards). However, the comparison remains largely anecdotal. The original comment (I agree with that comment) by reviewer 1 was aimed at understanding the degree of concordance among surveillance approaches. For example, were patterns of ARG richness and resistance classes consistent across qPCR, metagenomics, and cultured isolates? A brief quantitative summary (even descriptive rather than statistical) of agreement and disagreement among methods would strengthen the manuscript and better support the conclusion that the approaches provide complementary information.

We thank the reviewer for this helpful suggestion and agree that a semi-quantitative analysis would strengthen the manuscript. As suggested by the reviewer, we performed a semi-quantitative analysis of ARG richness, isolate resistance profile and drug class presence/absence for each surveillance approach, with the goal of assessing concordance between methods and sample types. As the methods employed in this study measure distinct aspects of AMR and differ in their detection breadth direct statistical between multiple approaches was no appropriate. Instead, ARG richness was calculated for both qPCR and metagenomics data by enumerating unique ARG variants for each sample type and normalising by the total number of detected ARG for each method. For cultured isolates, resistance profiles were summarised as resistance per cultured isolate for each sample type. Finally, drug class presence/absence was determined for qPCR, metagenomics and culture-based approaches. The results are presented in Figure 6, comprising ARG richness and isolate resistance profiles (Figure 6a), and a concordance heatmap of drug classes across sample types (Figure 6b). This analysis supports our conclusions that the three approaches are complementary, with each providing distinct but overlapping information of the

environmental resistome. We have added several paragraphs at lines 345-374 describing these finding to the results section as follows:

'To further explore concordance between surveillance approaches we performed a semi-quantitative summary of ARG richness, resistance profiles of cultured isolates and drug class presence/absence across qPCR, metagenomics and culture-based methods (**Figure 6**). Metagenomics detected greater ARGs richness compared to qPCR or cultured isolates, reflecting both its broader detection capacity (>6,000 ARGs), but also the potential inflation of ARG richness through detection of genes associated with background ecological processes including efflux pumps and regulatory function (**Figure 6a**). Although these methods are disparate which precludes direct statistical comparison, ARG richness showed broad concordance between qPCR and metagenomics, where wastewater showed the greatest ARG richness across both methods (Figure S3A). This finding suggests that for wastewater which contain human-associated and pathogenic taxa, targeted qPCR can capture ARG diversity comparable to those observed using metagenomics. By contrast, resistance profiles of cultured isolates showed less concordance with qPCR or metagenomics data, with resistance per isolate highest in urban park soil and water when compared to other samples. This results likely reflects that targeted isolation represents a subset of the entire bacterial community.

Drug class profiles were less concordant between methods for each sample (**Figure 6b**). Metagenomics detected the greatest number of drug classes overall, consistent with its broader detection capacity and greater number of reference genes in the CARD database compared to targeted qPCR or antibiotics used to test cultured isolates. For the drug classes that qPCR was able to detect, qPCR and metagenomics often detected the same drug classes across samples. Similarly, cultured-based profiles were limited by the antibiotic classes tested in the Sensititre assays. Penicillin beta-lactams, tetracyclines and cephalosporins showed the greatest concordance across approaches, with putative resistance detected by all three methods in multiple sample types. Complete concordance between methods was observed for cephalosporins in agricultural samples and urban park water, cephalosporins, tetracyclines and penicillin beta-lactams in waste water, penicillin beta-lactams in wetland samples and aminoglycosides, cephalosporins, penicillin beta-lactams and tetracyclines in Yarra River samples.'

Lines 211-212: Inflation of efflux genes (issue also raised by reviewer #2): The interpretation of fluoroquinolone resistance genes requires additional caution. Most of the genes listed here (adeF, marA, soxS, soxR, sdrM, rsmA, acrA, acrB, acrE, acrF, acrS, emrA, emrB, emrR, mdtH, patB) do not directly confer clinically significant fluoroquinolone resistance in the same way as plasmid-mediated determinants (e.g., qnr genes, aac(6′)-Ib-cr, qepA, oqxAB) or QRDR mutations in gyrA, gyrB, parC, and parE. Rather, many of these genes encode efflux pumps or regulatory systems that contribute to reduced susceptibility and may only result in clinically relevant resistance when combined with other resistance mechanisms. Several of these efflux genes are core chromosomal components that may be present in fully susceptible strains. In many cases, resistance is associated with altered expression resulting from mutations in regulatory genes (e.g., marR, acrR, soxR, ramR) rather than the mere presence of the genes themselves. Therefore, detection of these genes by CARD/RGI should be interpreted as putative resistance potential rather than evidence of fluoroquinolone resistance. This limitation should be acknowledged more explicitly in the Results and/or Discussion, particularly given the widespread fluoroquinolone-associated signals observed across sample types in Figures 2 and 3. The revised manuscript does acknowledges that many RGI hits represent intrinsic resistance determinants, stress-response systems, or efflux components. However, Figures 2 and 3 still treat all ARG classes similarly. As mentioned above for fluoroquinolones, aminoglycoside, and disinfectant-associated categories may also be heavily influenced by genes such as regulators and efflux systems. I suggest emphasizing that ARG abundance

should not be interpreted directly as AMR risk, particularly when many detected determinants likely represent intrinsic ecological functions.

We thank the reviewer for their important comments. We agree with the reviewer that interpretation of AMR risk in environmental settings is nuanced and requires caution. We have updated the text to more explicitly indicate that fluoroquinolone resistance detected in metagenomics data has the potential to contribute to decreased susceptibility, but will in most cases not confer full clinical resistance. We have also updated the text to further emphasize that abundance of these ARGs does not necessarily reflect AMR risk. We have also updated the Figure 2 and 3 to include 'putative resistance by drug class', and have revised fluoroquinolone, glycopeptide and disinfectant resistance in these figures to 'resistance-associated' in the figure key.

The results and discussion sections have been updated at lines 171-181, 217-227, 396-399 as follows:

'…However, this finding likely reflects the environmental role of the genes that confer resistance to these antibiotics, rather than an active AMR risk. Additionally, the abundance of fluoroquinolones, aminoglycoside, and disinfectant-associated categories are largely influenced by genes encoding regulators and efflux systems, which likely do not explicitly confer clinical resistance, but may contribute to reduced susceptibility. For example, putative aminoglycoside and disinfecting agent resistance appeared to be primarily due to the abundance of osmotic stress related response regulators (*kdpE*) and efflux pumps (*qacG*) (**Table S3**). It should be emphasised that caution should be taken when attributing ARG abundance to AMR risk, especially when many of the genes detected by RGI likely reflects intrinsic ecological processes rather than active resistance…'

'…Many of these genes encode transcriptional regulators, two component systems, or efflux pumps that are associated with reduced susceptibility and clinical AMR only when paired with other resistance mechanisms, therefore their contribution to clinical resistance should be approached with caution. These genes are also important for stress responses, and other ecological processes, and their abundance likely primarily reflects their importance for environmental survival, rather than active AMR risk. This was specifically observed for putative fluoroquinolone resistance where many of the genes identified are resistance-associated (*adeF, marA, soxS, soxR, sdrM, rsmA, acrA, acrB, acrE acrF, acrS, emrA, emrB, emrR, mdtH, patB)* rather than explicitly conferring resistance (*qnr genes, aac(6')-Ib-cr, qepA, oqxAB,* or mutations in *gyrA, gyrB, parC,* or *parE*)…'

'...Many of the highly abundant ARGs identified were resistance-associated rather than explicitly conferring resistance. These findings further highlight that ARG abundance on its own should not be interpreted as active AMR risk…'

Table S3: The reported abundance of rpoB2 deserves further clarification. Rifampicin resistance is generally mediated by specific mutations within the RNA polymerase β-subunit (rpoB) rather than by gene presence. The manuscript should clarify whether the detected sequences contained known resistance-conferring mutations or whether annotation was based solely on homology to resistance-associated rpoB alleles. This is particularly important because Table S8 reports a best sequence identity of only ~92%, which may not be sufficient to confidently infer a resistance phenotype.

We thank the reviewer for this comment, but we partially disagree in this instance. RGI-CARD identifies sequences that closely match to the *Bifidobacterium adolescentis rpoB* reported in Lokesh *et al.,* 2018 to confer intrinsic resistance to rifampicin. Compared to other organisms that are sensitive to rifampicin and acquire resistance through mutation in *rpoB*, *B. adolescentis* have species specific polymorphisms that confer intrinsic resistance to rifampicin. Additional mutations in the 81-bp rifampicin resistance determining region (RRDR) can enhance this baseline resistance in *Bifidobacterium*, but they are already intrinsically

resistant. Multiple alignment of our MAG *rpoB* hit amino acid sequences showed that they were similar to the Bifidobacterium *rpoB* homologue, and different to an intrinsically sensitive organism such as *Mycobacterium tuberculosis*. We have chosen not to modify the text in this case because rifampicin resistance was only identified in three MAGs which all showed similarity to the *B. adolescentis rpoB.*

Table S3: Consider adding a 'Drug Class' column before the 'Gene' column. This would make it easier for readers to interpret the table and relate individual ARGs to the resistance classes discussed throughout the manuscript.

We thank the reviewer for this suggestion and have updated Table S3 to include a ‘Drug Class’ column before the “Gene” column.

Table S8: Several resistance hits have low sequence identity., e.g., glycopeptide resistance hits, which are also based on individual van-cluster genes (e.g., vanT, vanW, vanY) detected at low sequence identity (~33-40%). Please clarify if complete or near-complete resistance operons were ever identified.

We thank the reviewer for calling attention to this important limitation. We agree that these putative glycopeptide resistance-associated hits in Table S8 were identified at relatively low sequence identity and correspond to individual genes within the van resistance cluster (e.g. *vanT*, *vanW* and *vanY*), rather than demonstrating the presence of the complete resistance operon. We have updated the text at line 227-234 to be more specific about limitations of interpreting these hits as evidence of glycopeptide resistance based solely on CARD/RGI detection as follows:

‘Furthermore, we noted that many glycopeptide-resistance-associated gene hits (*vanG, vanH, vanO, vanT, vanW, vanY*) showed between ~22-76% amino acid identity (**Table S8**) and were not always encoded as part of a *van* resistance cluster, suggesting that these ARGs likely do not confer resistance. Therefore, both putative glycopeptide and fluoroquinolone resistance should be interpreted cautiously in these environmental resistomes. This reinforces the idea that ARG abundance is not entirely indicative of AMR risk, particularly when AMR-associated determinants likely represent underlying ecological processes…’

The manuscript notes that an average of only 21.6% of metagenomic reads mapped back to recovered MAGs, with some samples showing up to 96% unmapped reads. While this is not unexpected for complex environmental communities, it highlights that ARG-host assignments derived from MAGs represent only a subset of the total community. It would be helpful if the authors briefly discussed which sample types exhibited the lowest MAG recovery and how this may influence interpretation of MAG-associated ARG profiles.

We agree with the reviewer’s comment, that ARG-host assignment based on MAGs is not representative of the entire resistome, we have therefore revised the text to highlight this point. We have examined MAG recovery across samples and have included a brief summary of the sample types that showed the highest and lowest levels of MAG recovery. Uneven MAG recovery likely reflects differences in community complexity and bacterial abundance across environmental sample types. ARGs identified within recovered MAGs therefore provide valuable information regarding the potential hosts and genomic context of resistance determinants, but do not capture ARGs associated with organisms that were not sufficiently abundant to be successfully assembled into MAGs. We have therefore clarified that MAG-associated ARG profiles should be interpreted as a partial representation of the resistome, with the extent of MAG recovery influencing the proportion of community-associated ARGs for which host assignments could be made (lines 246-253, 404-409):

'…Mapping of metagenomics reads back to MAGs showed an average of ~21.6% reads mapped, indicating that ARG-host assignments based on MAGs represent only a portion of the community. We also observed uneven MAG recovery between samples, likely reflecting differences in community complexity and bacterial abundance across samples. The agricultural sludge sample showed the lowest mapping overall (~96% unmapped), suggesting that much of the community remains uncaptured. By contrast, MAGs from the animal scats showed higher mapping of metagenomics reads (~43%), but still only captured a portion of the community....'

'…Additionally, mapping reads back to MAGs showed an average of 21.6%, suggesting that only a portion of the community was captured using this method, and therefore only a proportion of ARG-host assignments are reflected in this data. While MAGs provide valuable information regarding the potential hosts and genomic context of resistance determinants, they cannot capture ARGs associated with organisms that were not sufficiently abundant to be successfully assembled into MAGs…'

The reported sequencing depth is inconsistent. The Results indicate ~120 million paired reads per sample (Line 158), whereas the Methods report ~238 million reads per sample (line 574). Please clarify whether these values represent read pairs versus total reads.

We thank the reviewer for this comment. The average sequencing depth is ~120 million 'paired-end reads', we have replaced ~238 million total reads in the methods with this number. The specific number of reads generated for each sample have also been added to Table S12.

Line 427: "Overall, we observed a relatively low prevalence but high diversity of resistance determinants" would benefit from clarification of how "prevalence" is defined. Wastewater samples showed substantial ARG burdens, whereas other environments showed lower abundance. It may be more accurate to specify that prevalence was generally low outside wastewater-associated samples.

Thank you for this suggestion, we have updated the manuscript at lines 486-487 to indicate that prevalence was generally low outside of wastewater-associated samples as suggested by the reviewer.

Additionally, the limited number of samples/sites per environment restricts the ability to generalize these findings and should be acknowledged when drawing broader conclusions about environmental AMR patterns.

We thank the reviewer for this comment. The manuscript was previously updated with discussion of these limitations. We kindly direct the reviewer to discussion about the limited sample size in the discussion section at lines 467-469. We have added 'sites' to this sentence to capture both sample number and site diversity.

Reference:

Lokesh D, Parkesh R, Kammara R. *Bifidobacterium adolescentis* is intrinsically resistant to antitubercular drugs. Sci Rep. 2018 Aug 9;8(1):11897. doi: 10.1038/s41598-018-30429-2. PMID: 30093677; PMCID: PMC6085307.

Re: mSystems00741-26R1 (**Integrated cross-sectoral surveillance of antimicrobial resistance genotypes and phenotypes across disparate reservoirs**)

Dear Dr. Thomas D Watts:

Thanks for your attentive response to the previous reviewer comments. I have assessed the revised manuscript and see no major additional changes needed.

Please remember to make the data publicly accessible (SRA currently states that "record has not yet been released").

Please also address any grammatical errors (e.g., repetition of "macrolide resistance" on line 143, sentence fragment on line 167).

Your manuscript has been accepted, and I am forwarding it to the ASM production staff for publication. Your paper will first be checked to make sure all elements meet the technical requirements. ASM staff will contact you if anything needs to be revised before copyediting and production can begin. Otherwise, you will be notified when your proofs are ready to be viewed.

Data Availability: ASM policy requires that data be available to the public upon online posting of the article, so please verify all links to sequence records, if present, and make sure that each number retrieves the full record of the data. If a new accession number is not linked or a link is broken, provide production staff with the correct URL for the record. If the accession numbers for new data are not publicly accessible before the expected online posting of the article, publication may be delayed; please contact ASM production staff immediately with the expected release date.

Publication Fees: For information on publication fees and which article types have charges, please visit our website. We have partnered with Copyright Clearance Center (CCC) to collect author charges. If fees apply to your paper, you will receive a message from no-reply@copyright.com with further instructions. For questions related to paying charges through RightsLink, please contact CCC at ASM_Support@copyright.com or toll free at +1-877-622-5543. CCC makes every attempt to respond to all emails within 24 hours.

ASM Membership: Corresponding authors may join or renew ASM membership to obtain discounts on publication fees. Need to upgrade your membership level? Please contact Customer Service at Service@asmusa.org.

Embargo Policy: A press release may be issued as soon as the manuscript is posted on the mSystems Latest Articles webpage. The corresponding author will receive an email with the subject line "ASM Journals Author Services Notification" when the article is available online.

Cover Image Submissions: If you would like to submit a potential Cover Image, please email a file and a short legend to msystems@asmusa.org. Please note that we can only consider images that (i) the authors created or own and (ii) have not been previously published. By submitting, you agree that the image can be used under the same terms as the published article. Image File requirements: TIF/EPS, 7.5 inches wide by 8.25 inches tall (at least 2,250 pixels wide by 2,475 pixels tall), minimum 300 dpi resolution (600 dpi preferred), RGB, and no figure elements, e.g., arrows or panel labels. The legend should be a short description of the image, 1-2 sentences recommended. Please use the ASM Journals Cover Image Sizing Tool (https://journals.asm.org/pb-assets/html/asm-cover-sizing-tool.html) to ensure that your proposed cover image meets our size requirements.

Thank you for submitting your paper to mSystems.

Sincerely,
Erica Hartmann
Editor
mSystems
