# Supplementary material for: Integrated cross-sectoral surveillance of antimicrobial resistance genotypes and phenotypes across disparate reservoirs
Source: mSystems. 2026 Sep 28;11(10):e00741-26. doi: 10.1128/msystems.00741-26 (PMC13712752; doi:10.1128/msystems.00741-26)
Supplement: Supplementary file 2 [file msystems.00741-26-s0001.docx]

**Supplemental data**

**Supplementary table headings and legends:**

**Table S1. Sample metadata.** Soil, sediment, water and faecal samples were collected using sterile 50 mL falcon tubes in triplicate from each of the locations indicated.

**Table S2. Metagenomics drug class relative abundance.** Short-read metagenomics data from each sample type were screened using Resistance Gene Identifier (RGI) to profile resistomes. Data were collapsed into drug class and relative abundance was calculated.

**Table S3. Metagenomics ARG gene counts as determined by RGI.** Short read metagenomics data from each sample type were screened for ARG carriage using RGI. Counts for ARGs detected in at least one sample are listed.

**Table S4. Metagenome read mapping to MAGs and isolate genomes.** Short read metagenomics data from each sample was mapped to individual MAGs and isolate genomes to understand coverage within each sample.

**Table S5. Coverage and relative abundance of MAGs using CoverM.** CoverM was used to determine the coverage and relative abundance of each MAG in each sample type, and determine the remaining unmapped fraction of each sample.

**Table S6. MAG taxonomic classification using GTDB-Tk.** Taxonomic classification of each MAG was performed using GTDB-tk, completeness and contamination estimates are also included.

**Table S7. MAG and isolate genome virulence factor screen using metaVF.** To understand pathogenic potential, MAGs and isolate genomes were screened against the virulence factor database (VFDB) for virulence factors using metaVF.

**Table S8. MAG AMR screening using RGI.**

**Table S9. Isolate taxonomic assignments determined by Sanger sequencing of the 16S rRNA gene and GTDB-Tk classification of genome assemblies (GTDB release 214) and checkM2 completeness metrics.**

**Table S10. Isolate resistance phenotype and ARG carriage determined by comparison to combined database.**

**Table S11. Sample preparation.**

**Table S12. Metagenomics sequencing depth per sample**

**Supplementary figures:**

**Figure S1. Map of sample sites across Victoria, Australia.** a) wider sampling sites including agricultural run off and sludge collection at a dairy farm in Camperdown and wastewater at Western Treatment Plant. b) Zoomed view of eastern Melbourne sample sites.

**Figure S2.** **Plasmid-borne genes among environmental isolates.** Non-efflux categorised antibiotic resistance genes (ARGs) identified with a percent identity of at least 70% are shown in **a)**, while efflux genes are shown in **b)**. The graph depicts the total number of ARGs present in each isolate separated into chromosomally encoded, or plasmid associated (mobilisable, non-mobilizable, and unknown).

**Figure S3. Sampling workflow. 1)** Samples were collected from various environments and **2)** processed (see methods) and **3)** plated onto selective and differential media (Bile Esculin Agar, MacConkey Agar, Sheep Blood Agar) to preferentially isolate putative opportunistic pathogens before antibiotic resistance susceptibility testing. **4)** DNA was extracted from both raw samples and bacterial isolates. **5)** DNA samples from raw samples were tested for antibiotic resistance gene carriage via Qiagen qPCR array. **6)** Both raw sample and bacterial isolate DNA was subjected to whole genome sequencing to generate short read data that was subsequently assembled (genomes and metagenome-assembled genomes). **7)** Raw metagenome reads, MAGs and isolate genomes were screened for ARGs using RGI or a combined ARG database to characterize AMR burden.
